# Dimeric Pillar[5]arene as a Novel Fluorescent Host for Controllable Fabrication of Supramolecular Assemblies and Their Photocatalytic Applications

**DOI:** 10.1002/advs.202206897

**Published:** 2023-01-22

**Authors:** Kaiya Wang, Rongbo Zhang, Zejing Song, Kaituo Zhang, Xueqi Tian, Srikala Pangannaya, Minzan Zuo, Xiao‐Yu Hu

**Affiliations:** ^1^ College of Materials Science and Technology Nanjing University of Aeronautics and Astronautics Nanjing 211106 P. R. China

**Keywords:** aggregation‐induced emission, dimeric pillar[5]arene, photocatalysis, supramolecular assembly

## Abstract

A dimeric fluorescent macrocycle ***m*‐TPE Di‐EtP5** (meso‐tetraphenylethylene dimeric ethoxypillar[5]arene) is synthesized based on the *meso*‐functionalized ethoxy pillar[5]arene. Through the connectivity of two pillar[5]arenes by C=C double bond, the central tetraphenylethylene (TPE) moiety is simultaneously formed. The resultant bicyclic molecule not only retains the host–guest properties of pillararenes but also introduces the interesting aggregation‐induced emission properties inherent in the embedded TPE structure. Three dinitrile derivatives with various linkers are designed as guests (**G1**, **G2**, and **G3**) to form host–guest assemblies with ***m*‐TPE Di‐EtP5**. The morphological control and fluorescence properties of the assemblies are successfully realized. **G1** with a shorter alkyl chain as the linker completely threads into the cavities of the host. **G2**, due to its longer chain length, forms a linear supramolecular polymer upon binding to ***m*‐TPE Di‐EtP5**. **G3** differs from **G2** by possessing a bulky phenyl group in the middle of the chain, which can be further assembled with ***m*‐TPE Di‐EtP5** to form supramolecular layered polymer and precipitated out in solution, and can be efficiently applied to photocatalytic reactions.

## Graphical abstract

A dimeric fluorescent macrocycle ***m*‐TPE Di‐EtP5** is synthesized based on the *meso*‐functionalized ethoxy pillar[5]arene. Three dinitrile derivatives (**G1**, **G2**, and **G3**) with different linkers are designed as guests to form host–guest assemblies with ***m*‐TPE Di‐EtP5**. The assembly of ***m*‐TPE Di‐EtP5⊃G3** forms layered supramolecular polymer and is applied efficiently for photocatalysis in aqueous solution.

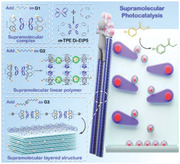

## 1 Introduction

The construction of novel macrocyclic hosts with luminescent properties has garnered great attention in the development of supramolecular functional materials.^[^ ^1^ ^]^ They not only possess unique molecular recognition properties, but also introduce luminescence properties into the self‐assembled systems. To date, there have been two main strategies for designing luminous macrocycles: one is to attach fluorophores to the edges of the macrocycles,^[^ ^2^ ^]^ which often leads to aggregation‐caused quenching (ACQ) effect;^[^ ^3^ ^]^ the other is to modify the macrocycles into aggregation‐induced emission luminogens (AIEgens),^[^ ^4^ ^]^ thus maintaining emission in the aggregated state. Recently, the latter has received increasing interest in the functionalization of fluorescent macrocyclic hosts.^[^ ^5^ ^]^

Pillararenes,^[^ ^6^ ^]^ as new generation of supramolecular macrocycles, are composed of hydroquinone units linked by methylene bridges. The symmetric pillar architecture and the easy accessibility to functionalization afford pillararenes with excellent host–guest properties.^[^ ^7^ ^]^ These features make them good candidates as host molecules in the fields of catalysis,^[^ ^8^ ^]^ fluorescence sensors,^[^ ^9^ ^]^ drug delivery,^[^ ^10^ ^]^ and molecular devices.^[^ ^11^ ^]^ The functionalization of the pillararene scaffold is of great interest as the introduction of substituents has a direct influence on their chirality, rigidity, and physical and chemical properties.^[^ ^12^ ^]^ So far, synthetic modifications have been extensively performed on the rims^[^ ^13^ ^]^ and, to a much lesser extent, at the methylene bridges (*meso* position).^[^ ^14^ ^]^ However, from a supramolecular point of view, this *meso* modification approach is more appealing as the additional binding sites or functional groups can be incorporated near the cavity without affecting the structural integrity and the host–guest properties of the pillararene skeleton.

**Scheme 1:**
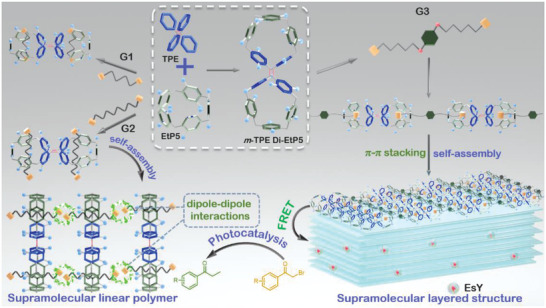
Schematic illustration of the controllable assembly of ***m*‐TPE Di‐EtP5** with three different guests and the photocatalytic process.

Recently, our group has reported a [1_5_]paracyclophane ([1_5_]PCP) dimer ***m*‐TPE Di‐[1_5_]PCP** ^[^ ^4c^ ^]^ by linking two *meso*‐functionalized [1_5_]PCP hosts. As one of the typical AIE materials, tetraphenylethylene (TPE)^[^ ^15^ ^]^ acts as a linker and endows it with fluorescent emission. Unfortunately, ***m*‐TPE Di‐[1_5_]PCP** does not possess the unique host–guest properties like pillararenes, and the fluorescence can only be regulated by metal ions. To address these shortcomings of the aforementioned host, we have designed and synthesized a novel host ***m*‐TPE Di‐EtP5**, in which a double bond as a linker was embedded between the two ethoxy pillar[5]arene (**Scheme** **1**). It is worth noting that ***m*‐TPE Di‐EtP5** is the very first dimeric pillararene that is artfully connected at the *meso* position. This bicyclic molecule not only retains the host–guest properties of pillararenes, but also confers excellent fluorescent properties due to the influence of TPE moiety embedded in the hosts.

To study the host–guest properties of ***m*‐TPE Di‐EtP5**, adiponitrile (**G1**) was first chosen as the guest molecule. X‐ray single crystal diffraction analysis of ***m*‐TPE Di‐EtP5**⊃**G1** revealed that each cavity of ***m*‐TPE Di‐EtP5** could fully bind one molecule of **G1**. Then, sebaconitrile (**G2**) with a longer alkyl chain was selected as the second guest molecule. **G2** could not gain full access into the cavities of ***m*‐TPE Di‐EtP5**, thus the cyano groups presented on the outside of the cavity. X‐ray single crystal diffraction of ***m‐*TPE Di‐EtP5**⊃**G2** revealed the formation of a linear supramolecular polymer due to the dipole–dipole interaction of adjacent –C≡N groups. The assembly of ***m*‐TPE Di‐EtP5**⊃**G3** (a guest molecule with bulky benzene in the middle of the chain) resulted in a white precipitate, which showed blue fluorescence under UV light. TEM experiment confirmed its lamellar polycrystalline‐like morphology. Owing to its insolubility and fluorescent properties, ***m*‐TPE Di‐EtP5**⊃**G3** assembly could be employed as a heterogeneous photocatalyst for the dehalogenation reaction with excellent yields in aqueous solution.

## 2 Results and Discussion

The synthetic route of ***m*‐TPE Di‐EtP5** is shown in Scheme S1 (Supporting Information). Ethoxy‐pillar[5]arene was synthesized as reported in the literature.^[^ ^16^ ^]^ First, monohydroxyl derivatization was performed at the *meso*‐position of the pillar[5]arene backbone using *N*‐bromosuccinimide (NBS).^[^ ^14b^ ^]^ The hydroxyl group was then oxidized to ketone by pyridinium dichromate (PDC). Finally, ***m*‐TPE Di‐EtP5** was obtained by McMurry coupling reaction. The synthetic details and characterization are shown in Figures S1–S5 (Supporting Information).

**Figure 1:**
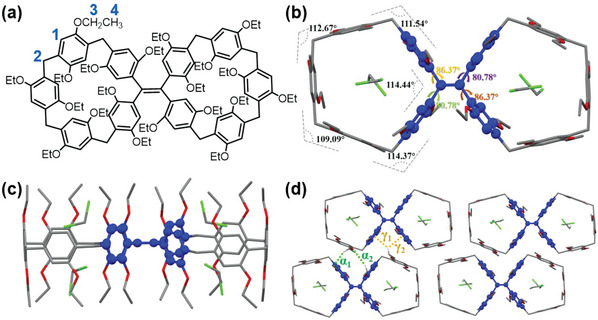
Chemical structure a) and single crystal structure of ***m*‐TPE Di‐EtP5** including top view b), side view c), and molecular packing form d). Yellow dashed lines represent C–H···*π* interactions (*γ*1 = 3.260 Å, *γ*2 = 3.232 Å); green dashed lines represent C–H···O interactions (*α*1 = 3.115 Å, *α*2 = 2.768 Å).

Single crystals suitable for X‐ray crystallographic analysis were collected by diffusion of *n*‐hexane into a dichloromethane solution of ***m*‐TPE Di‐EtP5** (**Figure** **1**). In the crystalline form, ***m*‐TPE Di‐EtP5** adopts a symmetrical rigid conformation, in which all ten diethoxyphenylene subunits are perpendicular to the molecular plane, forming a twin pillar[5]arene host (Figure 1a). The TPE moiety is embedded as a linker between two pillar[5]arenes, and the average bond angle of the cavities is about 112°. The two macrocycles of ***m*‐TPE Di‐EtP5** are almost in the same plane (Figure 1b,c) and are highly symmetrical. The crystal of ***m*‐TPE Di‐EtP5** is in a triclinic system, belonging to the P‐1 space group, in which the molecules are staggered and arranged in parallel (Figure 1d). Further analysis of the molecular packing revealed that interlayer C–H···*π* interactions with average atom–centroids distance of 3.246 Å exist between adjacent molecules involving the TPE and electron‐rich phenylene subunits. Multiple interlayer C–H···O interactions involving the TPE moiety are also present between neighboring molecules. These intermolecular interactions restrict the rotation of the intramolecular benzene ring, resulting in strong fluorescence emission of ***m*‐TPE Di‐EtP5** in the aggregated state (Figure 6b).

In order to verify the AIE properties of the bicyclic rings, the fluorescence of ***m*‐TPE Di‐EtP5** in a series of mixed solvents of chloroform and methanol was examined (Figure S12, Supporting Information). Chloroform was used as the good solvent while methanol as the poor one so that the aggregation of ***m*‐TPE Di‐EtP5** could be realized. The result showed that the fluorescence intensity of ***m*‐TPE Di‐EtP5** increased with the increase of methanol content, and the enhancement of fluorescence intensity was most obvious when the methanol content was 60%. Notably, in pure chloroform solution, the fluorescence intensity of ***m*‐TPE Di‐EtP5** was much higher than that of the monocyclic host **EtP5A‐DPE** ^[^ ^5c^ ^]^ and the unmodified host ***m*‐TPE Di‐[1_5_]PCP** (Figure S11, Supporting Information). In comparison with the monocyclic and unmodified hosts, the bicyclic structure of ***m*‐TPE Di‐EtP5** further restricts the intramolecular rotation (RIR)^[^ ^17^ ^]^ of the benzene ring in TPE moiety, exhibiting AIE emission in the low‐aggregation state.

**Figure 2:**
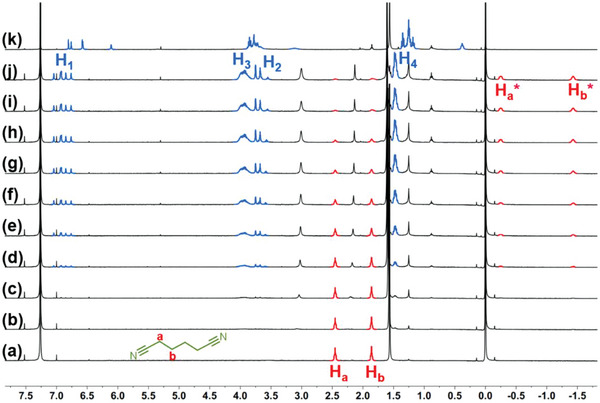
^1^H NMR spectra of **G1** (CDCl_3_, 400 MHz, and 298 K) in the presence of increasing equivalents of ***m*‐TPE Di‐EtP5**; from (a) to (j): 0, 0.025, 0.05, 0.1, 0.15, 0.2, 0.3, 0.4, 0.5, and 0.6 equivalents. k) free ***m*‐TPE Di‐EtP5**.

Adiponitrile (**G1**), a classic guest that has been frequently studied for the binding with pillar[5]arene,^[^ ^18^ ^]^ was initially selected as the guest molecule. ^1^H NMR titration experiments were performed (**Figure** **2**), in which the host ***m*‐TPE Di‐EtP5** was titrated at a fixed guest concentration. A slow exchange of this complex was observed on the NMR timescale. With the addition of ***m*‐TPE Di‐EtP5**, the ^1^H NMR signal peaks of the free **G1** protons (H_a_, H_b_) gradually disappeared and the bound guest signals appeared in the shielding region. After adding 0.5 equivalents of ***m*‐TPE Di‐EtP5**, the free **G1** signals disappeared completely, indicating each cavity of the host was occupied by one **G1** molecule.

**Figure 3:**
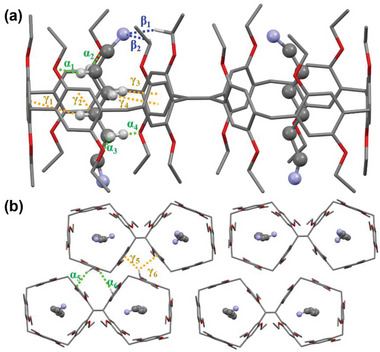
Single‐crystal X‐ray structure of ***m*‐TPE Di‐EtP5**⊃**G1**. a) Side view of ***m*‐TPE Di‐EtP5**⊃**G1**. b) Molecular packing form of ***m*‐TPE Di‐EtP5**⊃**G1**. Blue dashed lines represent C–H···N hydrogen bonds (*β*1 = 2.848 Å, and *β*2 = 2.778 Å), green dashed lines represent C–H···O hydrogen bonds (*α*1 = 3.206 Å, *α*2 = 3.193 Å, *α*3 = 3.084 Å, *α*4 = 3.254 Å, *α*5 = 3.171 Å, and *α*6 = 3.348 Å), and orange dashed lines represent C–H···*π* interactions (*γ*1 = 3.102 Å, *γ*2 = 3.045 Å, *γ*3 = 2.861 Å, *γ*4 = 2.960 Å, *γ*5 = 2.737 Å, and *γ*6 = 3.119 Å).

The formation of host–guest complex was further confirmed by X‐ray crystallographic analysis of a single crystal obtained by slow evaporation of its solution in dichloromethane/*n*‐hexane (**Figure** **3**). As expected, the guest **G1** fully threaded into each cavity of the host ***m*‐TPE Di‐EtP5**. Dipole–dipole forces play a significant role in the host–guest binding, while C–H···*π* and C–H···N/C–H···O interactions also jointly contribute to the complex formation and considerably reinforce the stability of the complex (Figure 3a). It is worth noting that the packing pattern of the host did not change since the guests were all present inside the host cavity (Figure 3b).

**Figure 4:**
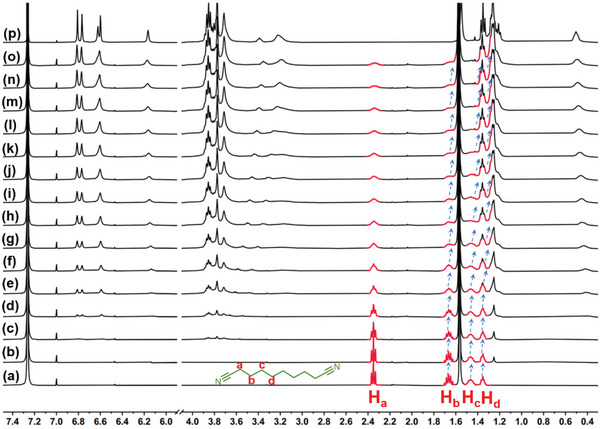
^1^H NMR spectra of **G2** (CDCl_3_, 400 MHz, and 298 K) in the presence of increasing equivalents of ***m*‐TPE Di‐EtP5**; from (a) to (o): 0, 0.1, 0.2, 0.3, 0.4, 0.5, 0.6, 0.7, 0.8, 0.9, 1.0, 1.1, 1.2, 1.4, and 1.6 equivalents. p) free ***m*‐TPE Di‐EtP5**.

In the case of guest **G2** with longer alkyl chain, ^1^H NMR titration experiment showed that its combination with ***m*‐TPE Di‐EtP5** was a fast exchange system. When ***m*‐TPE Di‐EtP5** was added dropwise at a fixed **G2** concentration (**Figure** **4**), the signal peaks of partial protons (H_b_, H_c_, and H_d_) on **G2** shifted to the upfield region, while no obvious chemical shift was observed for H_a_ adjacent to the cyano groups, suggesting that the cyano groups may exist outside the cavities. Therefore, we speculate that ***m*‐TPE Di‐EtP5**⊃**G2** will further assemble to form linear supramolecular polymers after forming interpenetrated complexes similar to **G1**. A broadening effect of the signal peaks was observed in variable concentration ^1^H NMR experiments of ***m***‐**TPE Di‐EtP5**⊃**G2** (Figure S7, Supporting Information); and the diffusion coefficients decreased by more than half with increasing concentration in the DOSY experiments (Figure S8, Supporting Information), indicating the formation of supramolecular polymers.

**Figure 5:**
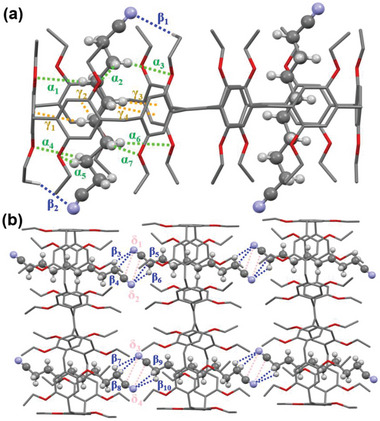
Single‐crystal X‐ray structure of ***m*‐TPE Di‐EtP5**⊃**G2**. a) Side view of ***m*‐TPE Di‐EtP5**⊃**G2**. b) Molecular packing form of ***m*‐TPE Di‐EtP5**⊃**G2**. Blue dashed lines represent C–H···N hydrogen bonds (*β*1 = 2.871 Å, *β*2 = 3.011 Å, *β*3 = 2.354 Å, *β*4 = 2.918 Å, *β*5 = 3.230 Å, *β*6 = 2.239 Å, *β*7 = 3.041 Å, *β*8 = 2.527 Å, *β*9 = 2.691 Å, *β*10 = 2.940 Å), green dashed lines represent C–H···O hydrogen bonds (*α*1 = 2.927 Å, *α*2 = 3.084 Å, *α*3 = 3.156 Å, *α*4 = 2.946 Å, *α*5 = 2.948 Å, *α*6 = 3.235 Å, *α*7 = 3.041 Å), orange dashed lines represent C–H···*π* interactions (*γ*1 = 2.994 Å, *γ*2 = 2.962 Å, *γ*3 = 2.894 Å, *γ*4 = 3.059 Å), and pink dashed lines represent dipole–dipole interactions (*δ*1 = 3.183 Å, *δ*2 = 3.259 Å, *δ*3 = 3.620 Å, *δ*4 = 3.495 Å).

The formation of linear supramolecular polymer was further confirmed by single crystal collected by diffusion of *n*‐hexane into a dichloromethane solution of ***m*‐TPE Di‐EtP5** and **G2** (**Figure** **5**). In contrast to **G1**, the cyano group of **G2** extended beyond the cavity, which provided structural support for its further assembly. The cyano groups of adjacent **G2** are close to each other and staggered (Figure 5b), forming a linear supramolecular polymer with dipole–dipole interaction as the main driving force. Simultaneously, the C–H···N interactions also jointly contribute to the polymer formation.

**Figure 6:**
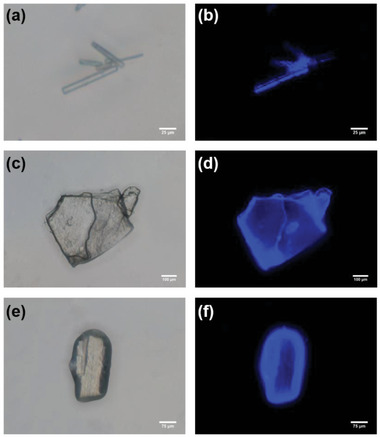
The images of the ***m*‐TPE Di‐EtP5** crystal a,b), ***m*‐TPE Di‐EtP5**⊃**G1** crystal c,d), and ***m*‐TPE Di‐EtP5**⊃**G2** crystal e,f) under bright field and UV‐light irradiation at the wavelength of 365 nm.

Furthermore, the fluorescence properties of free ***m*‐TPE Di‐EtP5** and its complex with **G1**/**G2** were investigated in both solution and solid state. When the powder of ***m*‐TPE Di‐EtP5** was subjected to grinding, the fluorescence intensity was enhanced (Figure S13, Supporting Information), and it shined even stronger in the crystal state (**Figure** **6**b), indicating the rearrangement of bicyclic rings upon grinding. It has been reported that **G1** can enhance the AIE emission of the monocyclic host **EtP5A‐DPE** (Figure S11a, Supporting Information) in solution but will quench its emission in the crystal state.^[^ ^5c^ ^]^ However, the bicyclic host ***m*‐TPE Di‐EtP5** upon binding to similar guest molecules exhibited completely different fluorescence emission properties compared to the monocyclic one. The addition of **G1** and **G2** quenched the fluorescence of the host in solution (Figures S14 and S15, Supporting Information), but preserved its fluorescence in crystals (Figure 6d,f). The rationale for this difference could be the fact that the guests existed only inside the cavity of ***m*‐TPE Di‐EtP5**, and did not change the packing mode of the host. In contrast, **G1** exists both inside and outside the cavity of the host in the crystal of **EtP5A‐DPE**⊃**G1**, thus changing the packing mode of **EtP5A‐DPE**. As a consequence, the presence of **G1** outside the cavity impeded the conjugation of TPE units between adjacent hosts, leading to the quenching of fluorescence.

**Figure 7:**
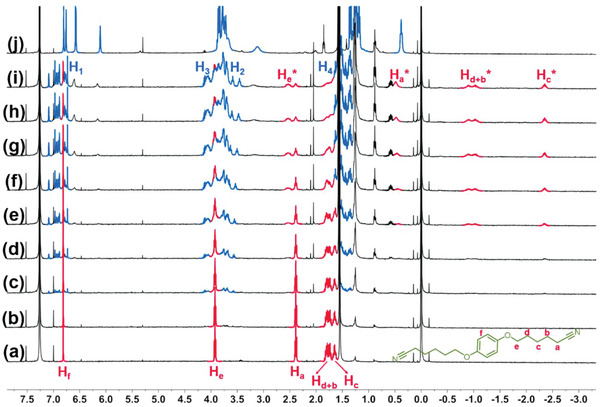
^1^H NMR spectra of **G3** (CDCl_3_, 400 MHz, and 298 K) in the presence of increasing equivalents of ***m*‐TPE Di‐EtP5**; from (a) to (i): 0, 0.025, 0.05, 0.1, 0.15, 0.2, 0.3, 0.4, 0.5, and 0.6 equivalents. j) free ***m*‐TPE Di‐EtP5**.

Subsequently, another guest **G3**, with a bulky phenyl group introduced in the middle of the linker was designed (Scheme S2 and Figure S6, Supporting Information), the structural variations resulted in different assembly mode and morphological features. ^1^H NMR titration experiments showed that the binding of ***m*‐TPE Di‐EtP5** and **G3** was slow exchange on the NMR time scale (**Figure** **7**). With the above titration experiments as well as ^1^H–^1^H COSY measurements (Figures S9 and S10, Supporting Information), it was found that the proton peaks (H_a_, H_b_, H_c_, H_d_, and H_e_) on the alkyl chain in **G3** showed upfield shifts and broaden effect with increasing host content, while the aromatic proton peaks (H_f_) exhibited no significant chemical shifts, indicating that the alkyl chains of **G3** were located in the shielding region inside the cavity of ***m*‐TPE Di‐EtP5**, while the phenyl group was located in the deshielding region outside the cavity. Based on the above results, we expect that the assembly mode of ***m*‐TPE Di‐EtP5** and **G3** may differ from that of **G1** and **G2**: each cavity of ***m*‐TPE Di‐EtP5** will bind to the alkyl cyanide portion of one side in **G3**, and the resultant assembly will be further assembled driven by the *π*–*π* stacking provided by the central benzene ring of **G3**, resulting in the formation of stacked supramolecular polymers.

**Figure 8:**
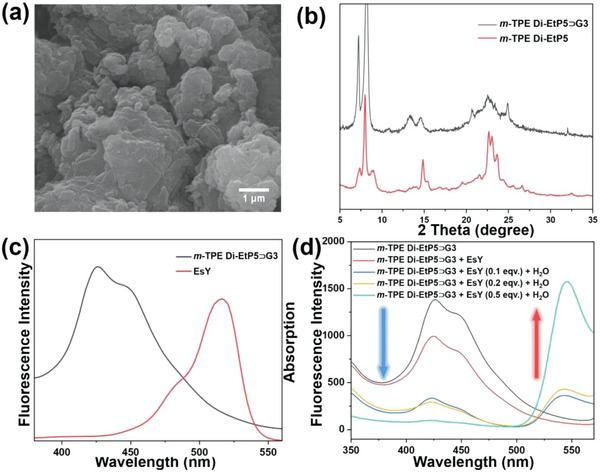
a) TEM image of ***m*‐TPE Di‐EtP5**⊃**G3**. b) PXRD patterns of ***m*‐TPE Di‐EtP5**⊃**G3** and ***m*‐TPE Di‐EtP5**. c) Normalized absorption and emission spectra of the EsY acceptor and the ***m*‐TPE Di‐EtP5**⊃**G3** donor assembly. d) Fluorescence spectra of the ***m*‐TPE Di‐EtP5**⊃**G3** assembly before and after addition of EsY.

The above speculation was confirmed by the fact that the ***m*‐TPE Di‐EtP5**⊃**G3** assembly was prone to precipitation in solution (Figure S17, Supporting Information). In addition, the precipitated solids cannot be redissolved in any solvents even upon heating and sonication. To further clarify the morphology of the precipitated solids, transmission electron microscopy (TEM) was first recorded (**Figure** **8**a), which revealed the formation of tightly stacked lamellar structure. The powder X‐ray diffraction (PXRD) patterns exhibited good crystallinity (Figure 8b).^[^ ^19^ ^]^ In comparison with the PXRD patterns of ***m*‐TPE Di‐EtP5** (Figure S19, Supporting Information), ***m*‐TPE Di‐EtP5**⊃**G3** showed great difference, which can be ascribed to the formation of the aggregates.

The obtained assembly of ***m*‐TPE Di‐EtP5**⊃**G3** retained excellent AIE properties (Figure S18. Supporting Information), making it an ideal donor for energy transfer in photocatalysis.^[^ ^20^ ^]^ Simultaneously, the insolubility and layered structure endow it with excellent adsorption properties. After the adsorption of eosin Y (EsY) as an acceptor, a nanoreactor mimicking natural photosynthesis was formed, which exhibited a high catalytic efficiency for the photocatalytic dehalogenation reaction for various bromoacetophenone derivatives in aqueous solution. Initially, the energy transfer between the ***m*‐TPE Di‐EtP5**⊃**G3** donor to the EsY acceptor in this composite system was examined via UV–vis and fluorescence spectroscopy, in which the absorption band of the

EsY acceptor showed good overlapping with the emission band of ***m*‐TPE Di‐EtP5**⊃**G3** donor (Figure 8c). To further illustrate the Förster resonance energy transfer (FRET) process, a solid‐state fluorescence characterization was carried out (Figure 8d). Upon the addition of EsY and subsequent grinding, the fluorescence intensity of ***m*‐TPE Di‐EtP5**⊃**G3** decreased, but no emission peak of EsY could be observed. However, when a trace amount of water was added to moisten the solid to simulate the state in solution, the fluorescence intensity of the ***m*‐TPE Di‐EtP5**⊃**G3** donor decreased significantly, while the emission peak of EsY at 540 nm increased significantly, indicating that efficient energy transfer occurred, and the energy transfer efficiency was calculated to be 90.3% (for details, see Figure S16 in the Supporting Information).

**Scheme 2:**
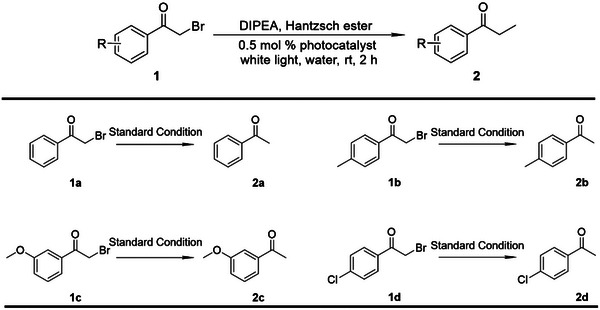
Dehalogenation reactions of different 2‐bromo‐1‐phenylethanone derivatives as substrates in the presence of ***m*‐TPE Di‐EtP5**⊃**G3**‐EsY under white light irradiation.

White light (20 W) was then used as a solar light simulator for the photocatalytic dehalogenation reactions.^[^ ^21^ ^]^ In the presence of 0.5 mol% ***m*‐TPE Di‐EtP5**⊃**G3**‐EsY in aqueous solution, 2‐bromo‐1‐phenylethanone (**1a**) gave acetophenone (**2a**) as the product in good yield under white light irradiation for 2 hours (**Scheme** **2**). The yield was obtained from ^1^H NMR spectra (Figure S20, Supporting Information) by relative method.^[^ ^22^ ^]^ To understand the role of the donor and acceptor, these catalytic reactions were carried out under different controlled conditions (**Table** **1**). In the absence of EsY (Figure S21, Supporting Information) or ***m*‐TPE Di‐EtP5**⊃**G3** (Figure S22, Supporting Information), the obtained product yield was very low under light irradiation. Notably, while under dark conditions (no light irradiation), there was hardly any product observed in the resulting solution. Subsequently, different bromoacetophenone derivatives modified with electron‐donating (**1b**, **c**) or electron‐withdrawing (**1d**) substituents were selected as substrates for the dehalogenation reactions. Most of substrates afforded the corresponding products in high yields (Figures S23–S25, Supporting Information), demonstrating the general applicability of ***m*‐TPE Di‐EtP5**⊃**G3**‐EsY as an efficient photocatalyst. It is worth noting that due to its insolubility, ***m*‐TPE Di‐EtP5**⊃**G3** could be easily recovered from the reaction solution after filtration and washing. After repeated recycling, ***m*‐TPE Di‐EtP5**⊃**G3** still maintained its high photocatalytic performance (Figure S26, Supporting Information).

**Table 1:** Dehalogenation reaction of 2‐bromo‐1‐phenylethanone under various reaction conditions

Entry	R	Photocatalyst^a)^	Light irradiation	Yield^b)^
1	H	None	Yes	28%
2	H	EsY	Yes	41%
3	H	***m*‐TPE Di‐EtP5**‐EsY	Yes	59%
4	H	***m*‐TPE Di‐EtP5**⊃**G3**	Yes	45%
5	H	***m*‐TPE Di‐EtP5**⊃**G3**‐EsY	No	<1%
6	H	***m*‐TPE Di‐EtP5**⊃**G3**‐EsY	Yes	99%
7	*p*‐Me	***m*‐TPE Di‐EtP5**⊃**G3**‐EsY	Yes	>99%
8	*m*‐OMe	***m*‐TPE Di‐EtP5**⊃**G3**‐EsY	Yes	>99%
9	*p*‐Cl	***m*‐TPE Di‐EtP5**⊃**G3**‐EsY	Yes	79%

^a)^ Reaction conditions: bromoacetophenone (20 mg, 0.1 mmol), Hantzsch ester (28 mg, 0.11 mmol), *N,N*‐diisopropylethylamine (DIPEA, 35 µL, 0.2 mmol), ***m*‐TPE Di‐EtP5**⊃**G3**‐EsY in water, 20 W white light, rt, N_2_, 2 h;

^b)^ Product yield was obtained from ^1^H NMR spectra.

To better understand the process for this photocatalytic dehalogenation reaction, a possible reaction mechanism was proposed in Figure S27 (Supporting Information) based on previous reports.^[^ ^21^, ^23^ ^]^ Initially, ***m*‐TPE Di‐EtP5**⊃**G3** donor absorbs light energy and changes from the ground state to the excited state (***m*‐TPE Di‐EtP5**⊃G3*) upon light irradiation. After accepting the energy transfer from ***m*‐TPE Di‐EtP5**⊃G3*, EsY*, which transitions from the ground to the excited state is reduced by Hantzsch ester to form the radical anion EsY^•−^. Subsequently, electrons are transferred from EsY^•−^ to the substrate *α*‐bromoacetophenone to generate the corresponding acetophenone radical, whilst EsY^•−^ is oxidized to EsY. Finally, the acetophenone radical combines with a hydrogen atom drawn from the radical cation of the Hantzsch ester to form acetophenone as the product. The Hantzsch ester is deprotonated in the presence of DIPEA and eventually becomes diethyl 2,6‐lutidine‐3,5‐dicarboxylate.

## 3 Conclusion

In conclusion, we have fabricated a novel type of pillar[5]arene dimer ***m*‐TPE Di‐EtP5**, in which a TPE moiety was embedded as a linker in the center of the molecule, thus making ***m*‐TPE Di‐EtP5** an AIEgen. While exhibiting the AIE effect, this molecule inherits the host–guest properties between pillararenes and dinitrile compounds. Its complexation with **G1** revealed that each cavity of the host is fully occupied by one guest molecule, which was confirmed by X‐ray single crystal diffraction. Subsequently, to construct supramolecular polymers, we selected sebaconitrile with longer chain as the guest **G2**. The binding of **G2** to the host molecule is similar to that of **G1**, but the extension of the cyano groups outside the host cavities leads to the formation of linear supramolecular polymers, which was also evidently observed by single crystal diffraction. To regulate the host–guest assembly pattern, with **G2** as the basis we designed the guest molecule **G3** by introducing a bulky phenyl group in the middle of the chain. The assembly of ***m*‐TPE Di‐EtP5**⊃**G3** showed both excellent fluorescence and insolubility. After the adsorption of eosin Y as an acceptor on the assembled surface of ***m*‐TPE Di‐EtP5**⊃**G3**, a nanoreactor mimicking natural photosynthesis was formed and exhibited high catalytic efficiency for the photocatalytic dehalogenation reaction. We believed that our methodology for constructing AIE macrocycles is inspirational has potential applications in photocatalysis, photodynamic therapy, chemical sensing and bioimaging in the future.

[CCDC 2154315, 2174232 and 2194663 contain the supplementary crystallographic data for this paper. These data can be obtained free of charge from The Cambridge Crystallographic Data Centre via www.ccdc.cam.ac.uk/data_request/cif.]

## Conflict of Interest

The authors declare no conflict of interest.

## Supporting information

Supporting Information

Supporting Information

## Data Availability

The data that support the findings of this study are available in the supplementary material of this article.

## References

[1] a) C. Zhang , Z. Wang , L. Tan , T. L. Zhai , S. Wang , B. Tan , Y. S. Zheng , X. L. Yang , H. B. Xu , Angew. Chem., Int. Ed. 2015, 54, 9244; 10.1002/anie.201502912 26089125

[2] a) R. Kumar , A. Sharma , H. Singh , P. Suating , H. S. Kim , K. Sunwoo , I. Shim , B. C. Gibb , J. S. Kim , Chem. Rev. 2019, 119, 9657; 31306015 10.1021/acs.chemrev.8b00605

[3] L. L.e Bras , C. Adamo , A. Perrier , ChemPhotoChem 2019, 3, 794.

[4] a) J. Luo , Z. Xie , J. W. Lam , L. Cheng , H. Chen , C. Qiu , H. S. Kwok , X. Zhan , Y. Liu , D. Zhu , B. Z. Tang , Chem. Commun. 2001, 1740; 10.1039/b105159h 12240292

[5] a) S. N. Lei , H. Cong , Chin. Chem. Lett. 2022, 33, 1493;

[6] T. Ogoshi , S. Kanai , S. Fujinami , T.‐a. Yamagishi , Y. Nakamoto , J. Am. Chem. Soc. 2008, 130, 5022. 18357989 10.1021/ja711260m

[7] a) T. Ogoshi , T. A. Yamagishi , Y. Nakamoto , Chem. Rev. 2016, 116, 7937; 27337002 10.1021/acs.chemrev.5b00765

[8] a) K. Wang , J. H. Jordan , K. Velmurugan , X. Tian , M. Zuo , X. Y. Hu , L. Wang , Angew. Chem., Int. Ed. 2021, 60, 9205; 10.1002/anie.202010150 32794352

[9] a) W. Cui , H. Tang , L. Xu , L. Wang , H. Meier , D. Cao , Macromol. Rapid Commun. 2017, 38, 1700161; 10.1002/marc.201700161 28524251

[10] a) S. Fu , Y. Zhang , S. Guan , Q. Huang , R. Wang , R. Tian , M. Zang , S. Qiao , X. Zhang , S. Liu , X. Fan , X. Li , Q. Luo , C. Hou , J. Xu , Z. Dong , J. Liu , ACS Appl. Mater. Interfaces 2018, 10, 14281; 29664280 10.1021/acsami.8b03534

[11] a) X. Chi , G. Yu , L. Shao , J. Chen , F. Huang , J. Am. Chem. Soc. 2016, 138, 3168; 26862921 10.1021/jacs.5b13173

[12] a) N. L. Strutt , H. Zhang , S. T. Schneebeli , J. F. Stoddart , Acc. Chem. Res. 2014, 47, 2631; 24999824 10.1021/ar500177d

[13] a) M. Xue , Y. Yang , X. Chi , Z. Zhang , F. Huang , Acc. Chem. Res. 2012, 45, 1294; 22551015 10.1021/ar2003418

[14] a) J. H. Wang , H. T. Feng , Y. S. Zheng , Chem. Commun. 2014, 50, 11407; 10.1039/c4cc05189k 25131632

[15] a) J. Mei , Y. Hong , J. W. Lam , A. Qin , Y. Tang , B. Z. Tang , Adv. Mater. 2014, 26, 5429; 24975272 10.1002/adma.201401356

[16] T. Ogoshi , K. Kitajima , T. Aoki , S. Fujinami , T. A. Yamagishi , Y. Nakamoto , J. Org. Chem. 2010, 75, 3268. 20397710 10.1021/jo100273n

[17] E. P. J. Parrott , N. Y. Tan , R. Hu , J. A. Zeitler , B. Z. Tang , E. Pickwell‐MacPherson , Mater. Horiz. 2014, 1, 251.

[18] a) X. Shu , S. Chen , J. Li , Z. Chen , L. Weng , X. Jia , C. Li , Chem. Commun. 2012, 48, 2967; 10.1039/c2cc00153e 22314976

[19] L. Liu , Y. Hu , S. Huang , Y. Jin , J. Cui , W. Gong , W. Zhang , Chem. Sci. 2021, 12, 13316. 34777750 10.1039/d1sc03680g PMC8528016

[20] a) W. J. Li , X. Q. Wang , D. Y. Zhang , Y. X. Hu , W. T. Xu , L. Xu , W. Wang , H. B. Yang , Angew. Chem., Int. Ed. 2021, 60, 18761; 10.1002/anie.202106035 34125487

[21] M. Neumann , S. Füldner , B. König , K. Zeitler , Angew. Chem., Int. Ed. 2011, 123, 981. 10.1002/anie.201002992 20878819

[22] Z. Bai , K. Velmurugan , X. Tian , M. Zuo , K. Wang , X. Y. Hu , Beilstein J. Org. Chem. 2022, 18, 429. 35529891 10.3762/bjoc.18.45 PMC9039527

[23] a) Z. J. Wang , S. Ghasimi , K. Landfester , K. A. I. Zhang , J. Mater. Chem. A 2014, 2, 18720;

