# Supplementary material for: Dimeric Pillar[5]arene as a Novel Fluorescent Host for Controllable Fabrication of Supramolecular Assemblies and Their Photocatalytic Applications
Source: Adv Sci (Weinh). 2023 Jan 22;10(9):2206897. doi: 10.1002/advs.202206897 (PMC10037968; doi:10.1002/advs.202206897)
Supplement: Supplementary file 1 — Supporting Information [file ADVS-10-2206897-s001.pdf]

# Supporting Information

# Dimeric Pillar[5]arene as a Novel Fluorescent Host for Controllable Fabrication of Supramolecular Assemblies and their Photocatalytic Applications

Kaiya Wang, Rongbo Zhang, Zejing Song, Kaituo Zhang, Xueqi Tian, Srikala Pangannaya, Minzan Zuo, and Xiao-Yu Hu*

College of Materials Science and Technology, Nanjing University of Aeronautics and Astronautics, Nanjing 211106 (China) E-mail: huxy@nuaa.edu.cn (X.-Y. Hu)

## Table of Contents

Experimental Procedures ....... S2

Results and Discussion ....... S2

1. Synthesis of host ***m*-TPE Di-EtP5** and guest **G3** ....... S2

  Synthesis of compound **EtP5** ....... S2

  Synthesis of compound **EtP5-OH** ....... S3

  Synthesis of compound **EtP5=O** ....... S3

  Synthesis of compound ***m*-TPE Di-EtP5** ....... S4

  Synthesis of guest molecule **G3** ....... S6

2. Host-guest $^1$H NMR experiments ....... S7

3. Fluorescence spectra of ***m*-TPE Di-EtP5** and ***m*-TPE Di-EtP5** after adding guests ....... S9

4. XRD experiments of ***m*-TPE Di-EtP5** before and after grinding ....... S12

5. Photocatalytic debromination reactions based on ***m*-TPE Di-EtP5⊃G3** assembly ....... S12

6. X-ray crystallography data ....... S16

References ....... S17

## Experimental Procedures

The commercially available reagents and solvents were either employed as purchased or dried according to procedures described in the literatures. Ethoxy pillar[5]arene was synthesized according to previous reported method.[1] All reactions were performed under nitrogen atmosphere unless otherwise stated. Analytical thin layer chromatography (TLC) was performed using 0.25 mm silica gel plates. Column chromatography was performed with silica gel (200-300 mesh) produced by Shanghai Titan Scientific Co., Ltd. All yields were given as isolated yields. $^{1}$H and $^{13}$C NMR spectra were recorded on a Bruker Avance 400 MHz spectrometer with internal standard tetramethylsilane (TMS) and solvent signals as internal references at 298 K, and the chemical shifts ($\delta$) were reported in ppm and coupling constant ($J$) values were given in Hz. 2D $^{1}$H-$^{1}$H COSY spectra were also recorded on a Bruker Avance 400 MHz spectrometer. 2D $^{1}$H-$^{1}$H DOSY spectra were recorded on a Bruker Avance 600 MHz spectrometer. High-resolution electrospray ionization mass spectra (HR-ESI-MS) were recorded on an Agilent 6540Q-TOF LCMS equipped with an electrospray ionization (ESI) probe operating in positive-ion mode with direct infusion. UV-visible spectra were recorded with a Shimadzu UV 1780 UV-Vis Spectrophotometer. Fluorescence spectra were recorded on a Gangdong SCI F-380 fluorescence spectrophotometer. Scanning electron microscope (SEM) investigations were carried out using a FEI Quanta FEG 250 instrument. The crystal structures were determined by single-crystal X-ray analysis. Data collections were performed using a Bruker Apex Smart CCD diffractometer. Powder X-ray diffraction (PXRD) data were collected on a Bruker D8 ADVANCE X-Ray diffractometer. Fluorescence photographs of the crystals were taken on a Nikon-Eclipse-Ti confocal microscope.

## Results and Discussion

### 1. Synthesis of host *m*-TPE Di-EtP5 and guest G3

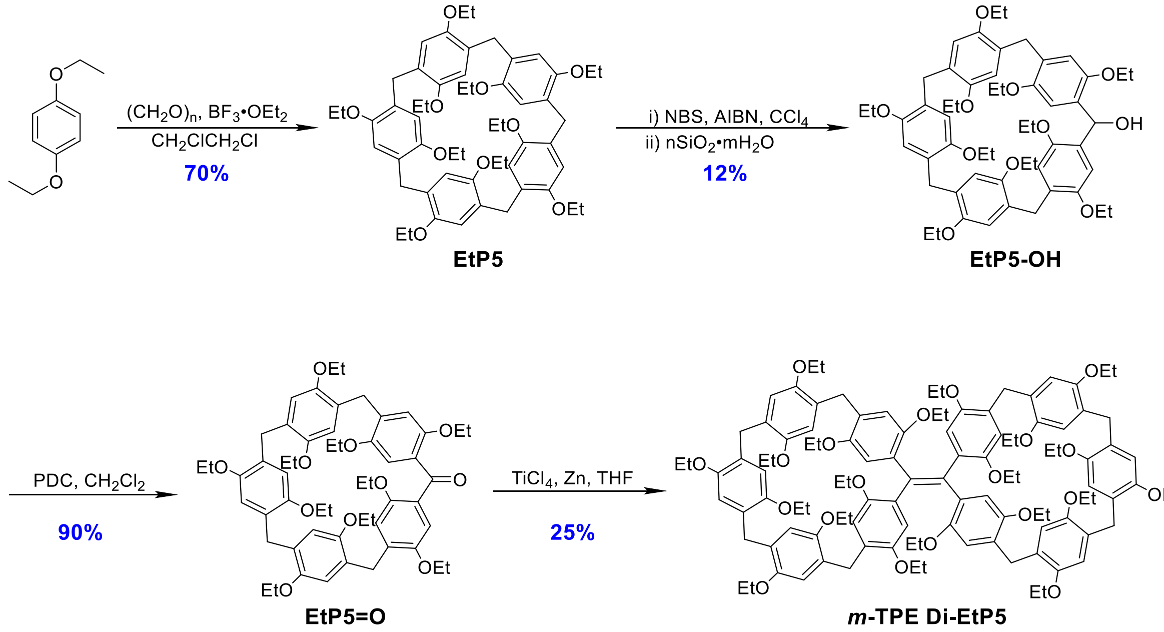


**Scheme S1** The synthetic route of ***m*-TPE Di-EtP5**.

### Synthesis of compound EtP5

The synthesis of **EtP5** was adapted from a previously reported method.[2] A solution of 1,4-diethoxybenzene (10 g, 60.0 mmol, 1.0 eq.) and $(CH_2O)_n$ (1.86 g, 60.0 mmol, 1.0 eq.) in 1,2-dichloroethane (400 mL) was cooled to 0 ˚C with ice bath for 10 min. Boron trifluoride diethyl etherate (7.5 mL, 72.0 mmol, 1.2 eq.) was then added and stirred for 1.5 h at room temperature, and the color of the solution gradually turned into dark blue. Then water (100 mL) was added to quench the reaction. After suction filtration, separation and drying, the organic phase was then concentrated. The crude product was purified by silica gel chromatography using petroleum ether/dichloromethane (2/1, *v/v*) as the eluent to give compound **EtP5** (7.0 g, 42.0 mmol, 70%) as a white solid. $^{1}$H NMR (400 MHz, $CDCl_3$, 298 K) $\delta$ (ppm): 6.74 (s, 10H), 3.86-3.81 (q, $J$ = 6.9 Hz, 20H), 3.76 (s, 10H), 1.28 (t, $J$ = 7.0 Hz, 30H).

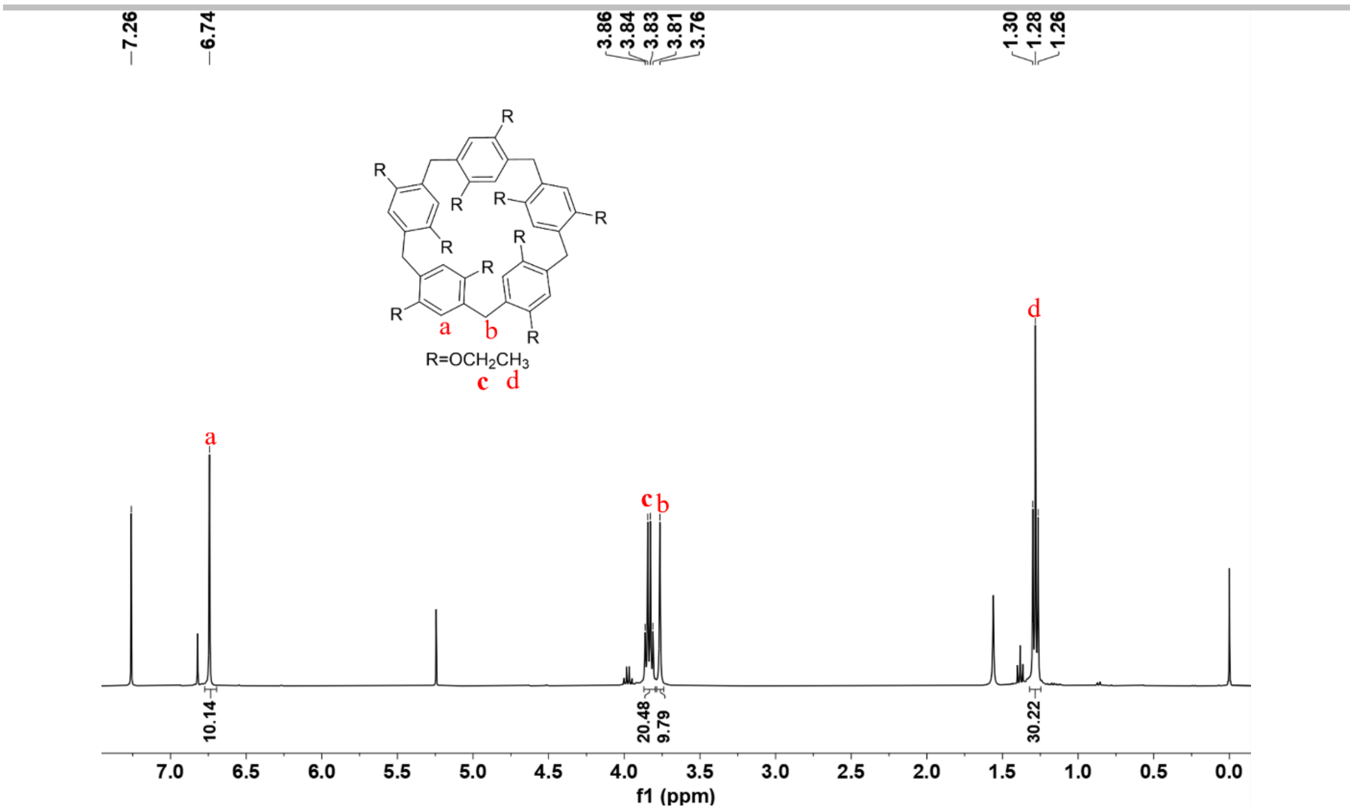


**Figure S1** $^1$H NMR spectrum of compound **EtP5** ($CDCl_3$, 400 MHz, 298 K).

### Synthesis of compound EtP5-OH

The synthesis of mono-functionalized **EtP5-OH** was adapted from a previously reported method.[2] To a solution of **EtP5** (6 g, 6.74 mmol, 1.0 eq.) in $CCl_4$ (180 mL), *N*-Bromosuccinimide (NBS, 0.64 g, 4.044 mmol, 0.6 eq.) and 2,2'-azobis (2-methylpropionitrile) (AIBN, 0.03 g, 0.183 mmol, 0.02 eq.) were added under the protection of nitrogen atmosphere. The mixture was stirred for 15 mins at room temperature before reflux, and the color of the solution gradually turned into light yellow. After about 2 h, another portion of NBS (0.3 g, 2.02 mmol, 0.3 eq.) was added. After about 2 h, another portion of NBS (0.3 g, 2.02 mmol, 0.3 eq.) was added again and the reaction was proceeded overnight. Silica gel was added and the color of the solution changed to dark black. The solution was then concentrated. The crude product was purified by silica gel chromatography using petroleum ether/dichloromethane (1/8, *v/v*) as the eluent to give compound **EtP5-OH** (0.70 g, 0.808 mmol, 12%) as a white solid. Since **EtP5-OH** is difficult to purify, we have improved the synthetic route to obtain the crude product by preliminary purification, which was directly used in the next step.

### Synthesis of compound EtP5=O

The crude product **EtP5-OH** (1 g, 1.1 mmol, 1.0 eq.) and pyridinium dichromate (PDC, 1.24 g, 3.3 mmol, 3.0 eq.) were dissolved in dichloromethane (20 mL). The mixture was stirred and heated to reflux for 3 h. The resulting solution was filtered over celite and concentrated. Column chromatography (petroleum ether/dichloromethane = 1/8, *v/v*) gave compound **EtP5=O** (900 mg, 0.99 mmol, 90%) as a white solid. Melting point: 164 °C. $^1$H NMR (400 MHz, $CDCl_3$, 298 K) $\delta$ (ppm): 6.94 (s, 2H), 6.86 (s, 2H), 6.81 (s, 2H), 6.56 (s, 2H), 5.91 (s, 2H), 3.95-3.84 (m, 16H), 3.80 (s, 8H), 2.87 (q, $J$ = 7.0 Hz, 4H), 1.42-1.30 (m, 22H), 1.24 (t, $J$ = 7.0 Hz, 8H).

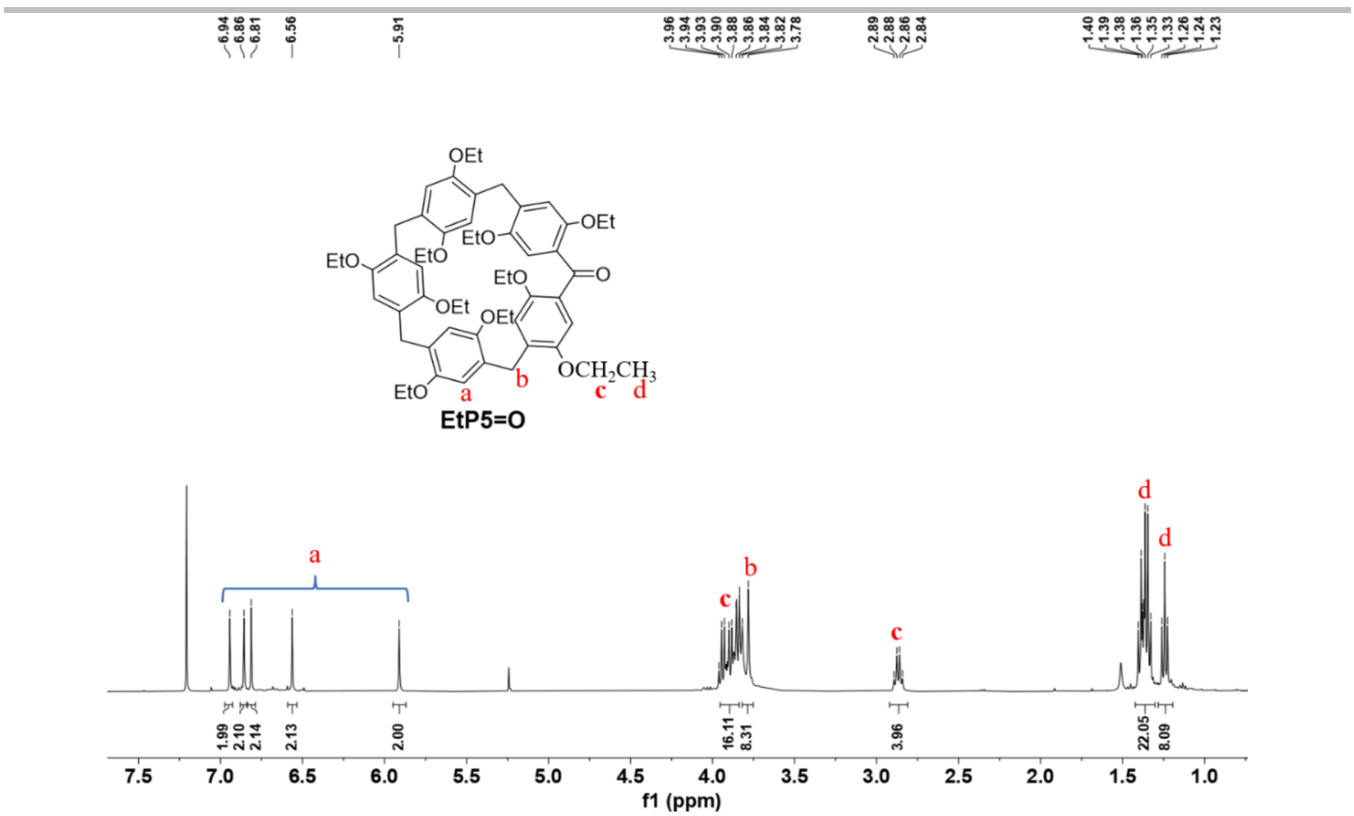


**Figure S2** $^{1}H$ NMR spectrum of compound **EtP5=O** ($CDCl_3$, 400 MHz, 298 K).

### Synthesis of compound *m*-TPE Di-EtP5

A 50 mL pressure-resistant bottle was charged with zinc powder (114.9 mg, 1.77 mmol, 8.0 eq.), compound **EtP5=O** (200 mg, 0.221 mmol, 1.0 eq.), and anhydrous tetrahydrofuran (5 mL) under $N_2$ protection. The mixture was cooled to –17 ˚C for 30 mins. Then $TiCl_4$ (0.2 ml, 1.77 mmol, 4.0 eq.) was added. After stirring for 1 h, the solution was heated to reflux overnight. The reaction was quenched by saturated $NaHCO_3$ solution (10 mL). After filtration, the filtrate was extracted with dichloromethane. The organic phase was dried over anhydrous $Na_2SO_4$ and the solvent was removed under vacuum. Finally, ***m*-TPE Di-EtP5** (55 mg, 0.055 mmol, 25%) was obtained by column chromatography (petroleum ether/dichloromethane = 1/1, *v/v*). Melting point: 218.3 °C. $^{1}H$ NMR (400 MHz, $CDCl_3$, 298 K) $\delta$ (ppm): 6.81 (s, 4H), 6.76 (s, 4H), 6.57 (d, $J$ = 4.3 Hz, 8H), 6.11 (s, 4H), 3.90-3.75 (m, 32H), 3.70 (d, $J$ = 16.8 Hz, 16H), 3.11 (s, 8H), 1.35 (t, $J$ = 7.0 Hz, 13H), 1.25 (td, $J$ = 7.0, 3.7 Hz, 25H), 1.22-1.15 (m, 12H), 0.38 (s, 10H). $^{13}C$ NMR (100 MHz, $CDCl_3$, 298 K) $\delta$ (ppm): 150.00, 149.01,129.51, 129.15, 128.72, 117.66, 115.43, 115.27-114.48, 63.71, 63.56-63.29, 29.09, 15.32-14.78. HR-ESI-MS: Calculated $[M+Na]^+$: $C_{110}H_{136}O_{20}Na^+$ = 1799.9522, Found: 1799.9450.

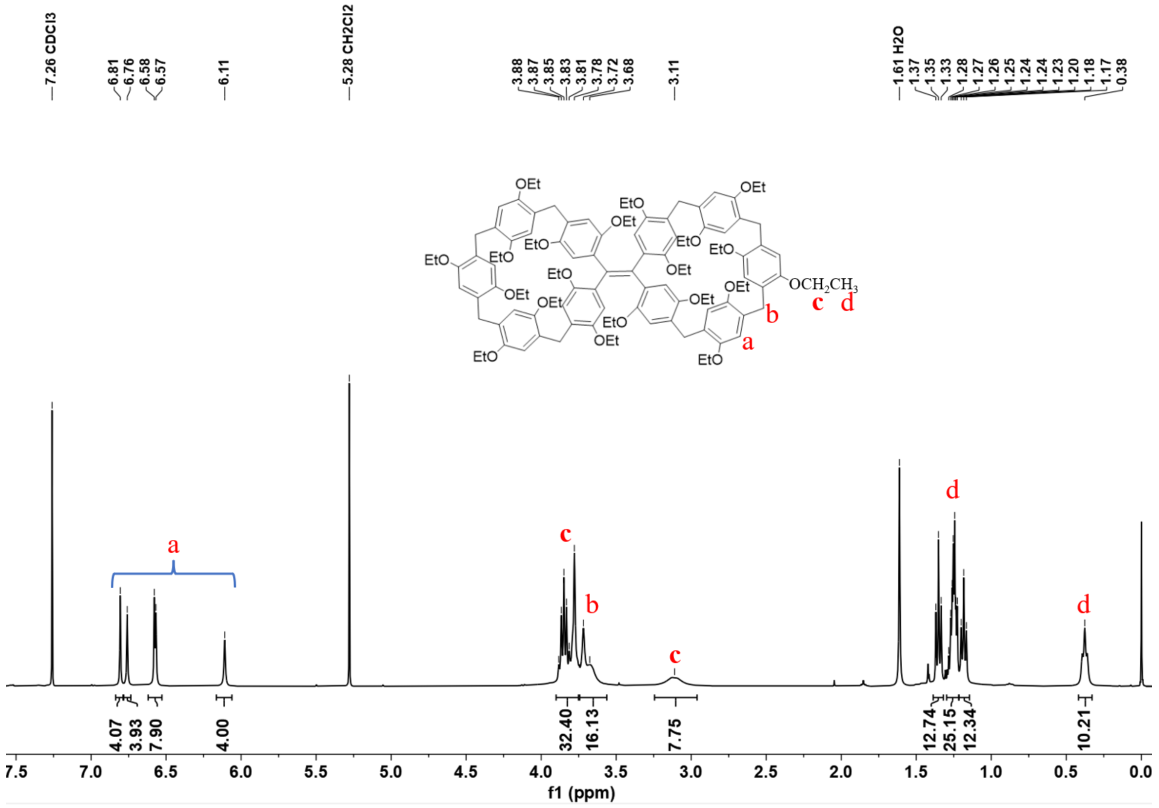


**Figure S3** $^{1}$H NMR spectrum of compound ***m*-TPE Di-EtP5** ($CDCl_3$, 400 MHz, 298 K).

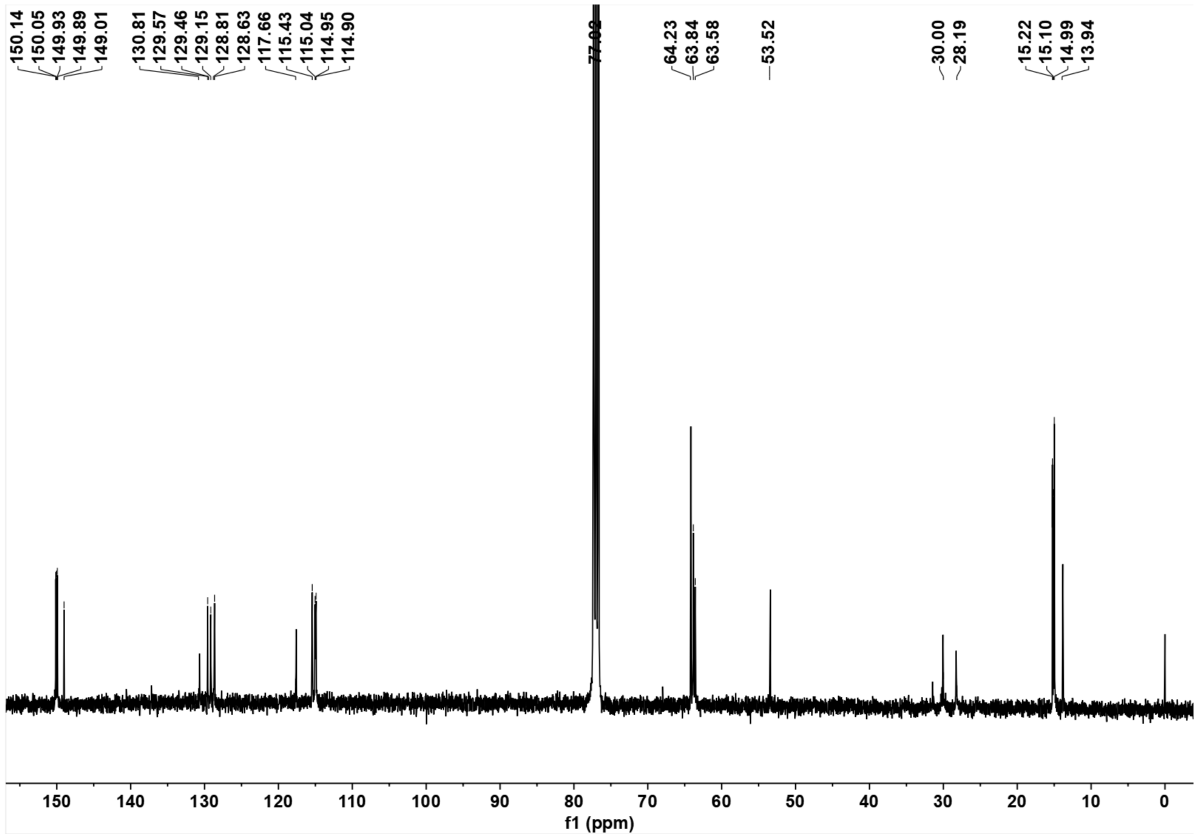


**Figure S4** $^{13}$C NMR spectrum of compound ***m*-TPE Di-EtP5** ($CDCl_3$, 100 MHz, 298 K).

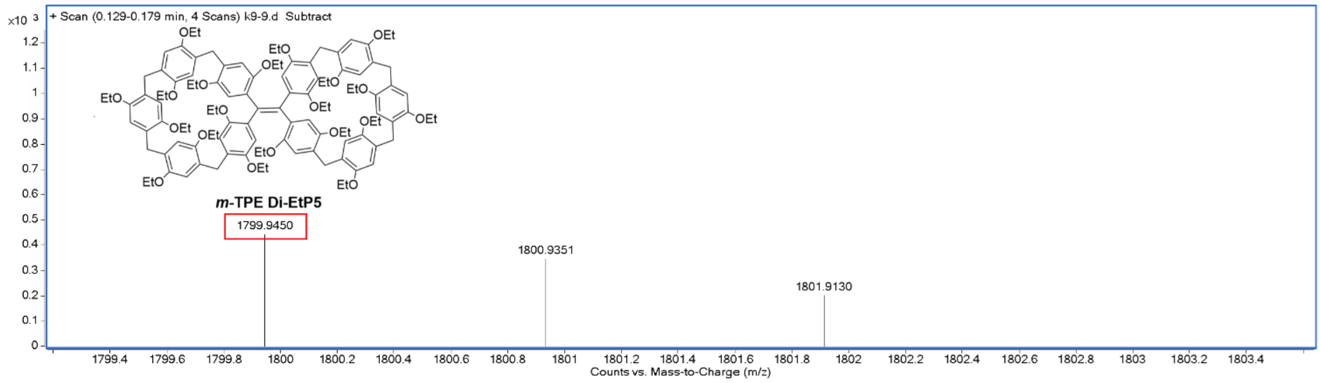


**Figure S5** HR-ESI-MS data for ***m*-TPE Di-EtP5** (Calculated $[M+Na]^+$: $C_{110}H_{136}O_{20}Na^+$ = 1799.9522, Found: 1799.9450).

## Synthesis of guest molecule G3

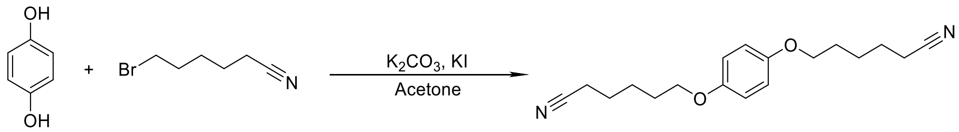


**Scheme S2** The synthetic route of compound **G3**.

The synthesis of **EtP5** was adapted from a previously reported method.[3] Hydroquinone (104.6 mg, 0.95 mmol, 1 eq.) and $K_2CO_3$ (981 mg, 7.10 mmol, 7.5 eq.) were dissolved in anhydrous acetone (5 mL) with heating and stirring for 1 h. Then, 6-bromocapronitrile (500 mg, 2.84 mmol, 3 eq.) was added into the above mixture. The mixture was refluxed overnight. When the reaction mixture was cooled to room temperature, the inorganic salt was filtered and washed with dichloromethane for several times. The organic phase was dried over anhydrous $Na_2SO_4$, filtered, and concentrated. The crude product was purified by silica gel chromatography (dichloromethane/petroleum ether = 5:2, *v/v*) to afford compound **G3**. $^1H$ NMR (400 MHz, $CDCl_3$, 298 K) $\delta$ (ppm): 6.81 (s, 4H), 3.92 (t, *J* = 6.2 Hz, 4H), 2.38 (t, *J* = 7.0 Hz, 4H), 1.86 – 1.70 (m, 8H), 1.70 – 1.59 (m, 4H).

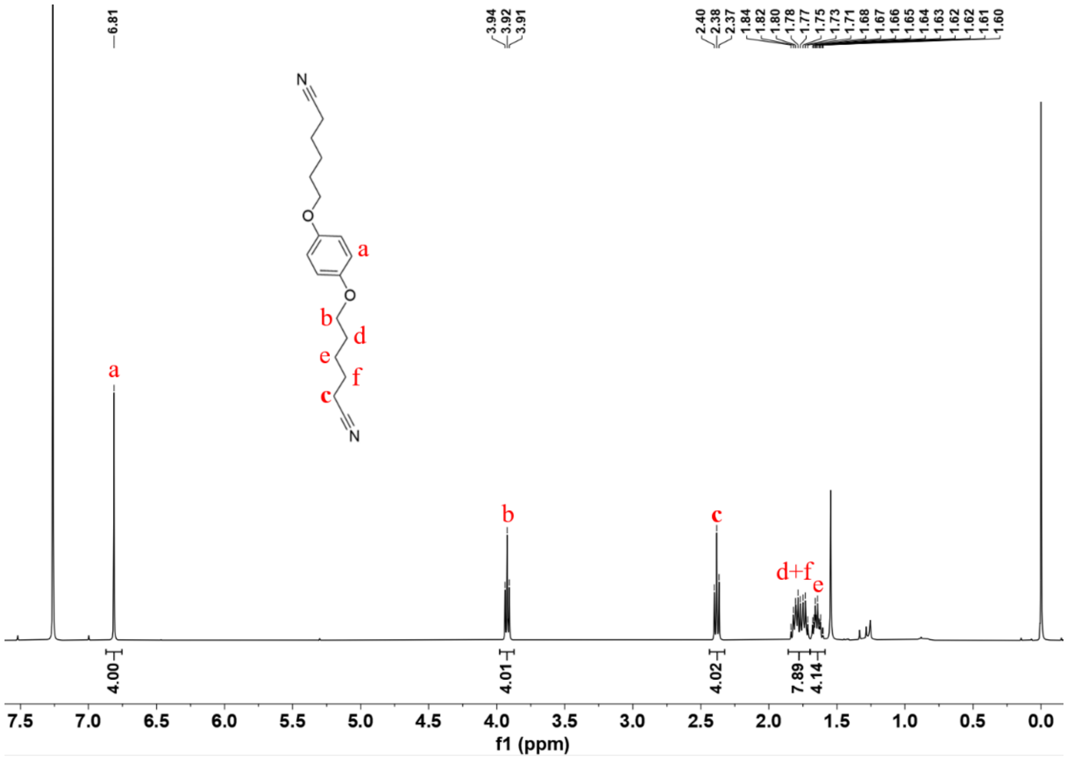


**Figure S6** $^1H$ NMR spectrum of compound **G3** ($CDCl_3$, 400 MHz, 298 K).

## 2. Host-guest $^1$H NMR experiments

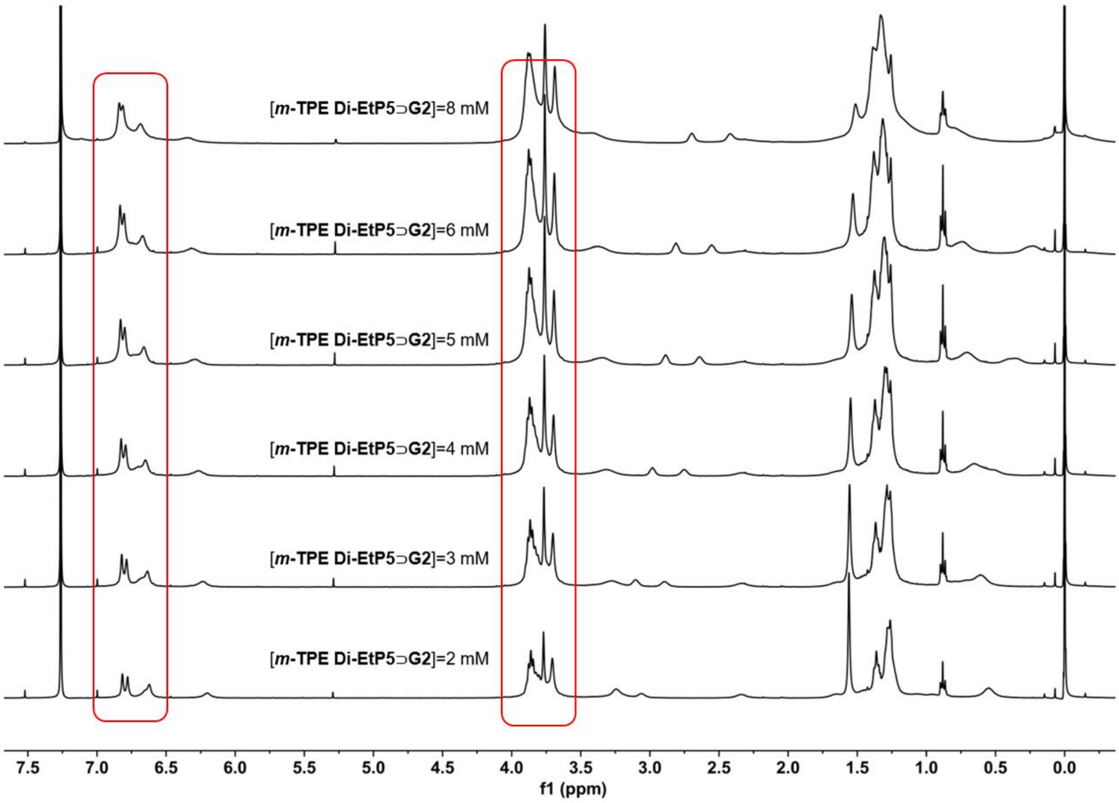


**Figure S7** Variable concentration $^1$H NMR ($CDCl_3$, 400 MHz, 298 K) spectra of ***m*-TPE Di-EtP5⊃G2** ([***m*-TPE Di-EtP5**]:[**G2**] = 1:2).

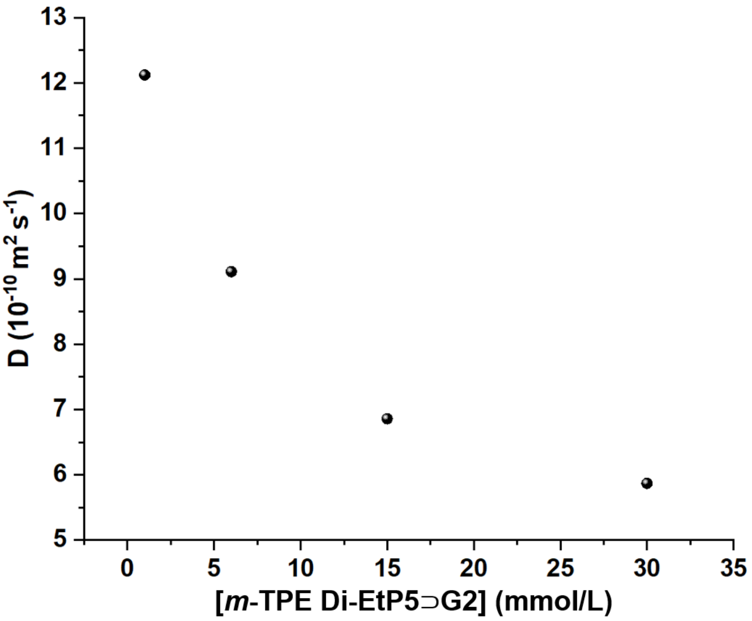


**Figure S8** Concentration dependence of diffusion coefficient D (600 MHz, $CDCl_3$, 298 K) of ***m*-TPE Di-EtP5⊃G2** ([***m*-TPE Di-EtP5**]:[**G2**] = 1:2).

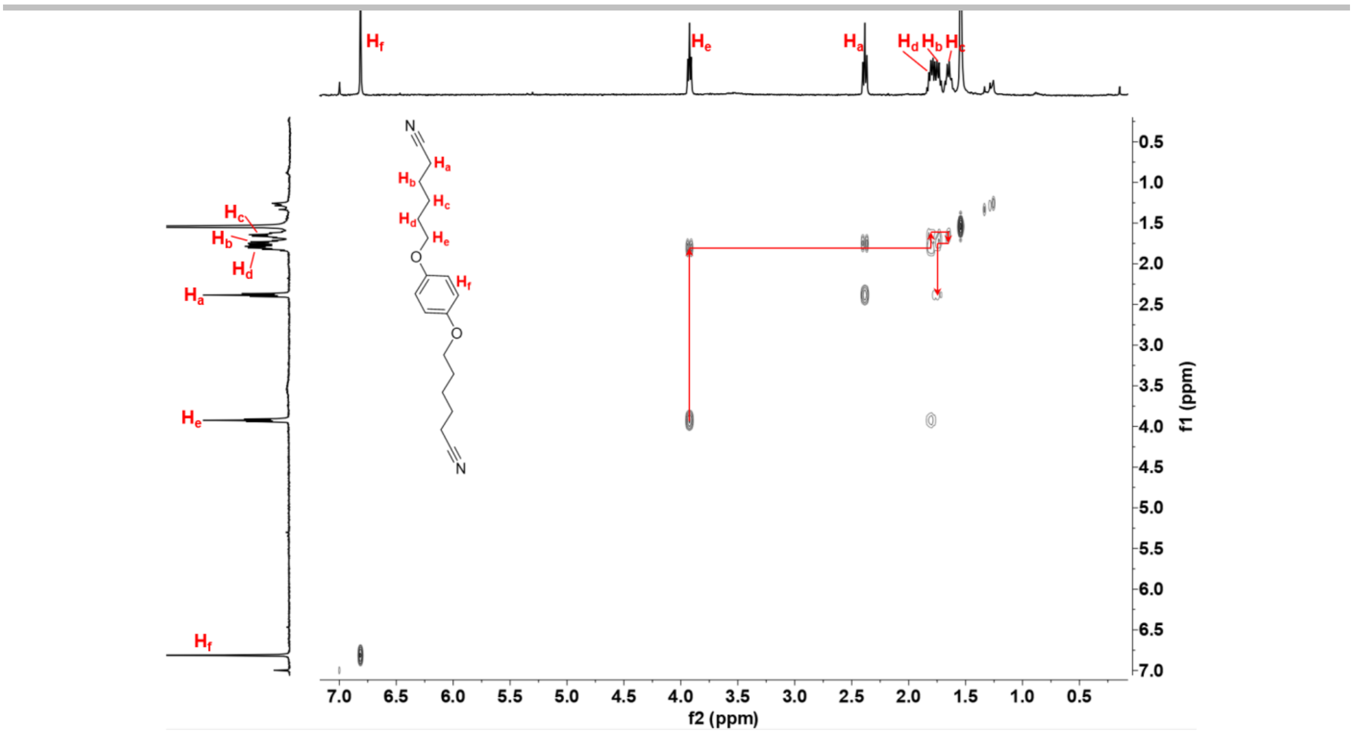


**Figure S9** Partial 2D $^{1}H$-$^{1}H$ COSY ($CDCl_3$, 400 MHz, 298 K) spectrum of **G3** ($1 \times 10^{-3}$ M).

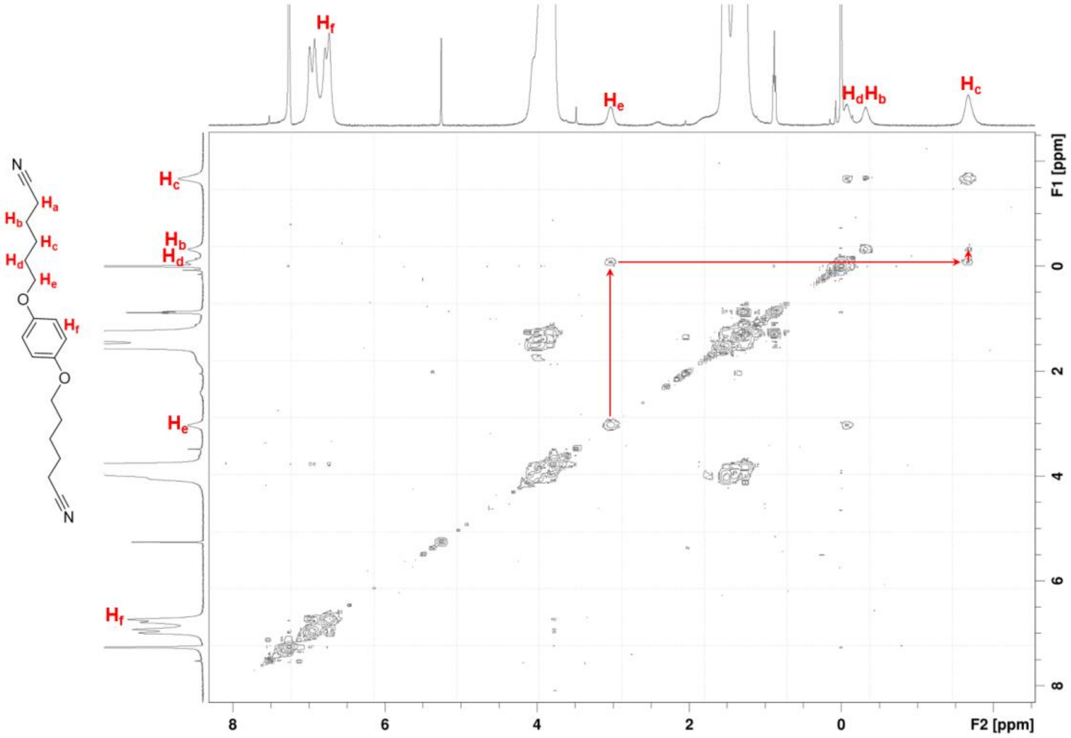


**Figure S10** Partial 2D $^{1}H$-$^{1}H$ COSY ($CDCl_3$, 400 MHz, 298 K) spectrum of **G3** ($1 \times 10^{-3}$ M) binding to template host **EtP5** ($2 \times 10^{-3}$ M). Due to the precipitation of ***m*-TPE Di-EtP5⊃G3** assembly, **EtP5** (ethoxy pillar[5]arene) was chosen as the template host.

## 3. Fluorescence spectra of *m*-TPE Di-EtP5 and *m*-TPE Di-EtP5 after adding guests

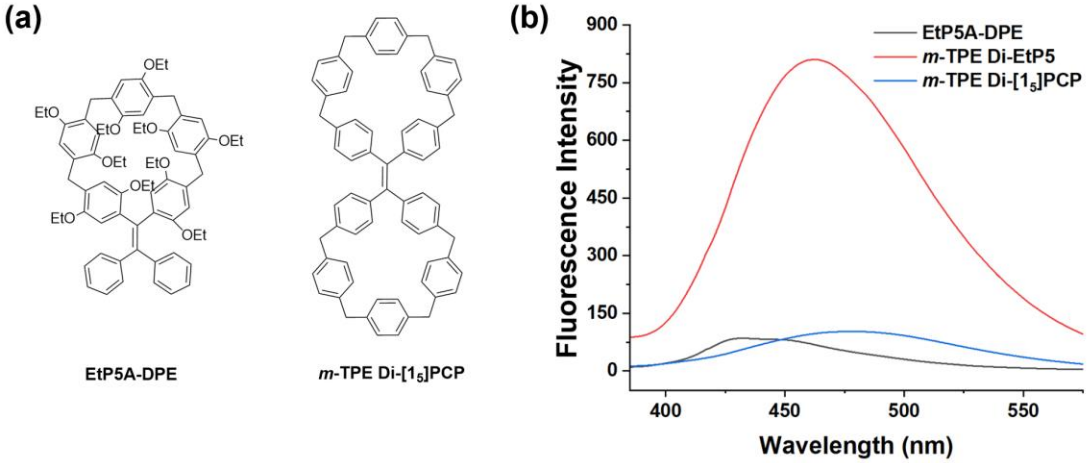


**Figure S11** (a) Chemical structure of **ETP5A-DPE** and ***m*-TPE Di-[1$_5$]PCP**. (b) Fluorescence spectra of ***m*-TPE Di-EtP5** (1 × 10$^{-5}$ M), **ETP5A-DPE** (1 × 10$^{-5}$ M) and ***m*-TPE Di-[1$_5$]PCP** (1 × 10$^{-5}$ M) in $CHCl_3$.

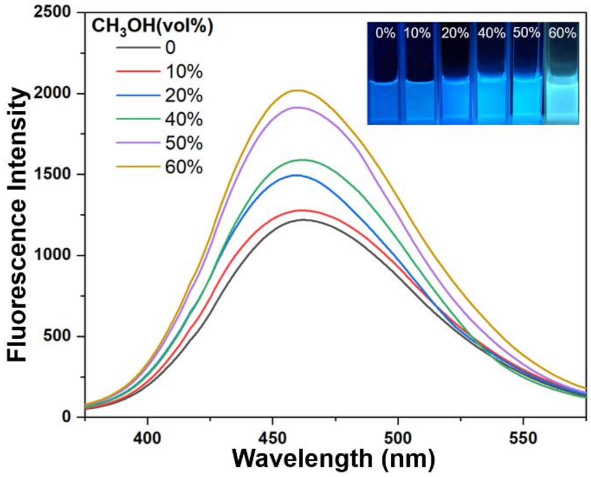


**Figure S12** Fluorescence spectra of ***m*-TPE Di-EtP5** (1 × 10$^{-5}$ M) in chloroform/methanol mixture with different methanol fractions upon UV light (300 nm) irradiation. Inset: fluorescence images of ***m*-TPE Di-EtP5** in corresponding solutions.

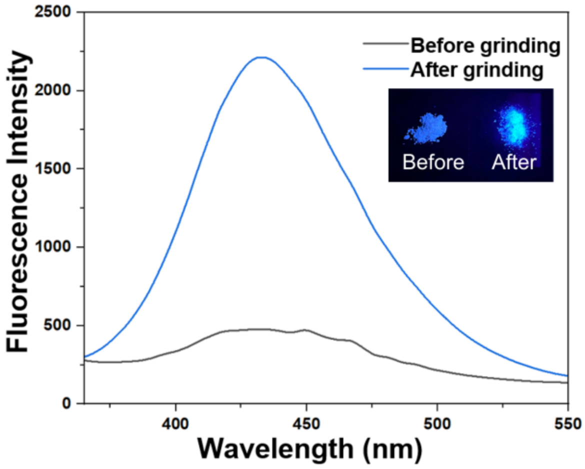


**Figure S13** Fluorescence spectra of ***m*-TPE Di-EtP5** before and after grinding upon UV light (300 nm) irradiation. Inset: fluorescence images of ***m*-TPE Di-EtP5** before and after grinding.

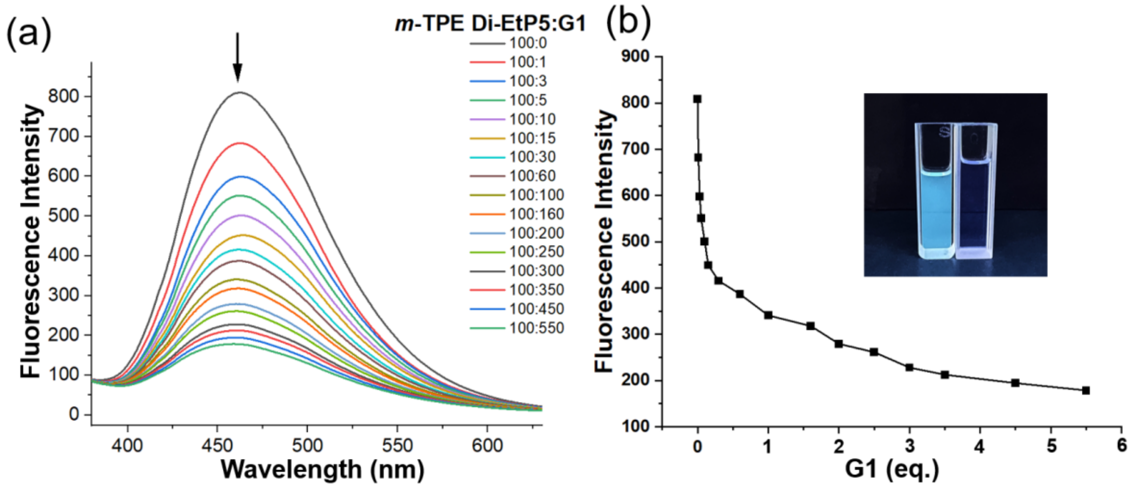


**Figure S14** (a) Fluorescence spectra of ***m*-TPE Di-EtP5** ($1 \times 10^{-5}$ M) with different equivalents of **G1** in $CHCl_3$. (b) Plot of the fluorescence intensity of ***m*-TPE Di-EtP5** ($1 \times 10^{-5}$ M) with different equivalents of **G1** in $CHCl_3$. Inset: fluorescence images of ***m*-TPE Di-EtP5** ($1 \times 10^{-5}$ M) and ***m*-TPE Di-EtP5** after adding 2 equivalents of **G1** in $CHCl_3$.

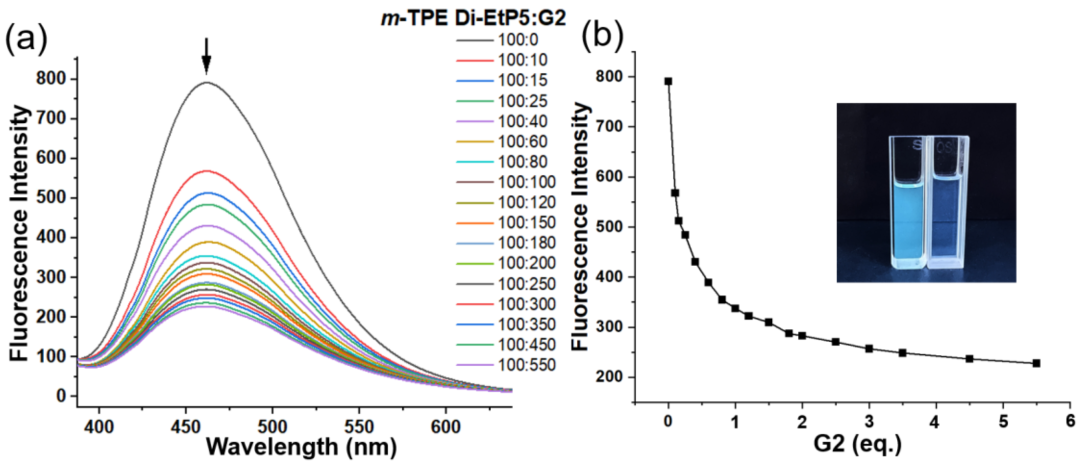


**Figure S15** (a) Fluorescence spectra of ***m*-TPE Di-EtP5** ($1 \times 10^{-5}$ M) with different equivalents of **G2** in $CHCl_3$. (b) Plot of the fluorescence intensity of ***m*-TPE Di-EtP5** ($1 \times 10^{-5}$ M) with different equivalents of **G2** in $CHCl_3$. Inset: fluorescence images of ***m*-TPE Di-EtP5** ($1 \times 10^{-5}$ M) and ***m*-TPE Di-EtP5** after adding 2 equivalents of **G2** in $CHCl_3$.

The energy transfer efficiency ($\Phi_{ET}$) was calculated using equation S1:

$\Phi_{ET} = 1 - I_{DA} / I_D$ (eq. S1)

Where $I_{DA}$ and $I_D$ are the fluorescence intensities of the emission of nanoparticles (donor and acceptor) and nanoparticles (donor), respectively when excited at 300 nm. And the energy transfer efficiency was calculated to be 90.3%.

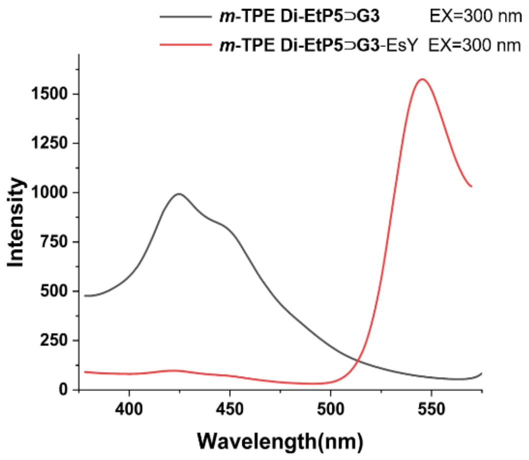


**Figure S16**. Fluorescence spectra of ***m*-TPE Di-EtP5⊃G3** and ***m*-TPE Di-EtP5⊃G3**-EsY. $\lambda_{ex}$ = 300 nm (***m*-TPE Di-EtP5⊃G3** = 30 mg, EsY = 15 mg).

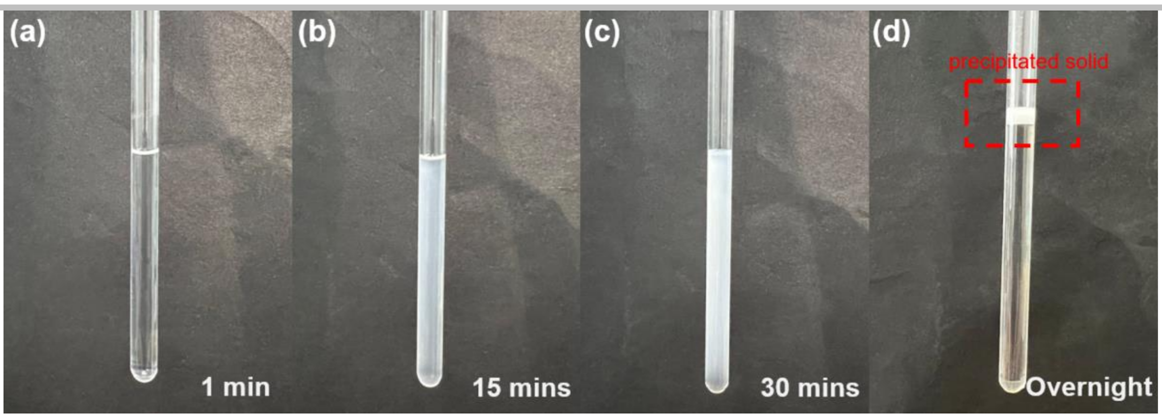


**Figure S17** The aggregation process of ***m*-TPE Di-EtP5⊃G3**: images of ***m*-TPE Di-EtP5** (1 × $10^{-3}$ M) after adding 1 equiv. of **G3** in $CDCl_3$. After the addition of **G3** for 1 min (a), 15 mins (b) and 30 mins (c), the ***m*-TPE Di-EtP5⊃G3** assembly gradually precipitated from the solution, and the precipitated solid suspended on the liquid surface overnight (d).

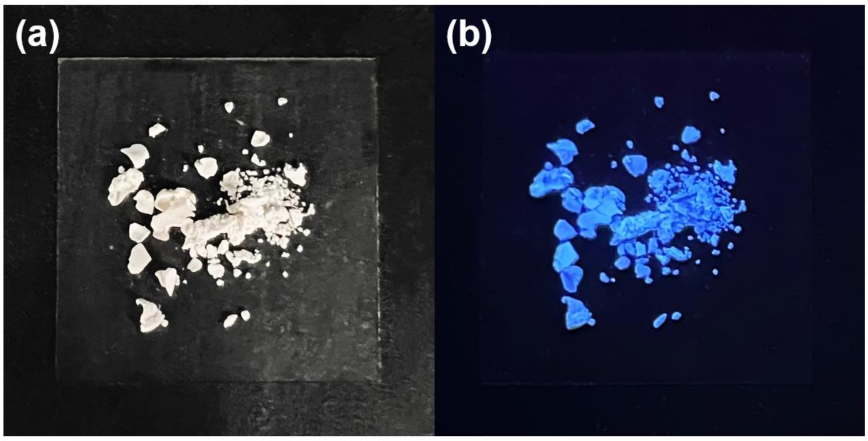


**Figure S18** The images of precipitated solid of ***m*-TPE Di-EtP5⊃G3** under bright field (a) and UV-light irradiation at the wavelength of 365 nm (b).

## 4. XRD experiments of *m*-TPE Di-EtP5 before and after grinding

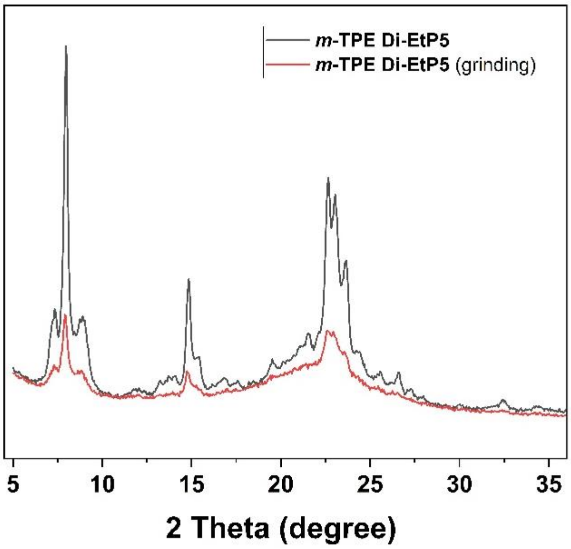


**Figure S19** The XRD images of ***m*-TPE Di-EtP5** before and after grinding.

## 5. Photocatalytic debromination reactions based on *m*-TPE Di-EtP5⊃G3 assembly

**Table S1** Dehalogenation reaction of 2-bromo-1-phenylethanone under various reaction conditions.

| Entry | R | Photocatalyst[a] | Light irradiation | Yield[b] |
|---|---|---|---|---|
| 1 | H | None | Yes | 28% |
| 2 | H | EsY | Yes | 41% |
| 3 | H | ***m*-TPE Di-EtP5**-EsY | Yes | 59% |
| 4 | H | ***m*-TPE Di-EtP5⊃G3** | Yes | 45% |
| 5 | H | ***m*-TPE Di-EtP5⊃G3**-EsY | No | <1% |
| 6 | H | ***m*-TPE Di-EtP5⊃G3**-EsY | Yes | 99% |
| 7 | *p*-Me | ***m*-TPE Di-EtP5⊃G3**-EsY | Yes | >99% |
| 8 | *m*-OMe | ***m*-TPE Di-EtP5⊃G3**-EsY | Yes | >99% |
| 9 | *p*-Cl | ***m*-TPE Di-EtP5⊃G3**-EsY | Yes | 79% |

[a] Reaction conditions: Bromoacetophenone (20 mg, 0.1 mmol), Hantzsch ester (28 mg, 0.11 mmol), *N*,*N*-diisopropylethylamine (DIPEA, 35 μL, 0.2 mmol), ***m*-TPE Di-EtP5⊃G3**-EsY in water, 20 W white light, rt, $N_2$, 2 h. [b] Product yield was obtained from $^1H$ NMR spectra.

Since ***m*-TPE Di-EtP5⊃G3** is insoluble in any solvents, $CDCl_3$ was selected to extract the product and substrate from the reaction solution after the completion of reaction. In this way, it contains the characteristic peaks of Hantzsch ester and DIPEA, but we only selected the -$CH_3$ group of the product and the -$CH_2$ group of the substrate for calibration. And relative measurements were used to calculate yields: $\eta = \frac{\frac{A1}{n1}}{\frac{A1}{n1}+\frac{A2}{n2}}\%$.

Here in Figure S20, $A_1$ is the integral area of the product -$CH_3$ group, $n_1$ is the number of protons in the characteristic peak of the product, $A_2$ is the integral area of the substrate -$CH_2$ group, $n_2$ is the number of protons in the characteristic peak of the substrate.

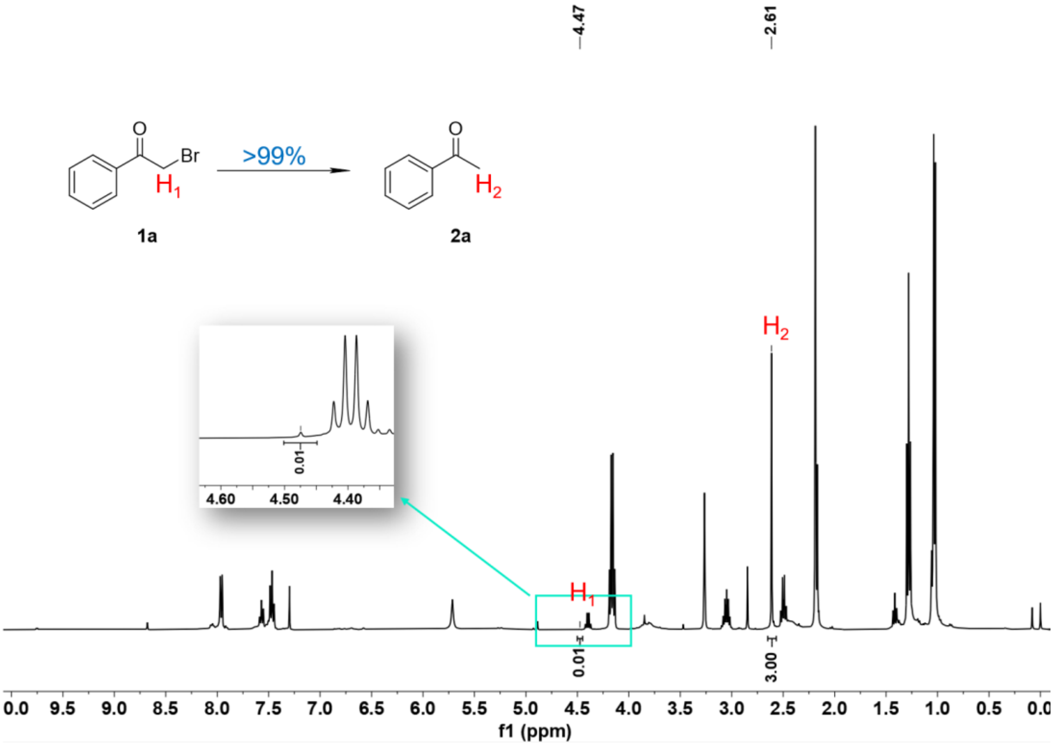


**Figure S20** $^1H$ NMR (400 MHz, $CDCl_3$, 298 K) spectrum of the reaction mixture of entry 6.

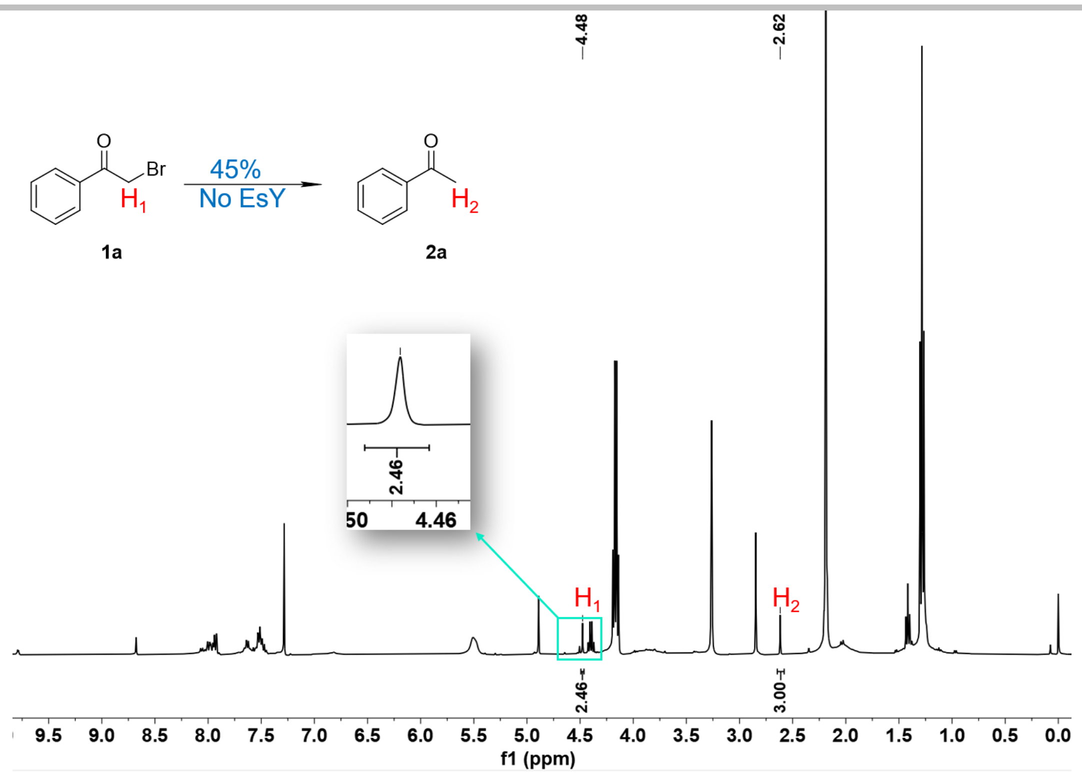


**Figure S21** $^1H$ NMR (400 MHz, $CDCl_3$, 298 K) spectrum of the reaction mixture of entry 4.

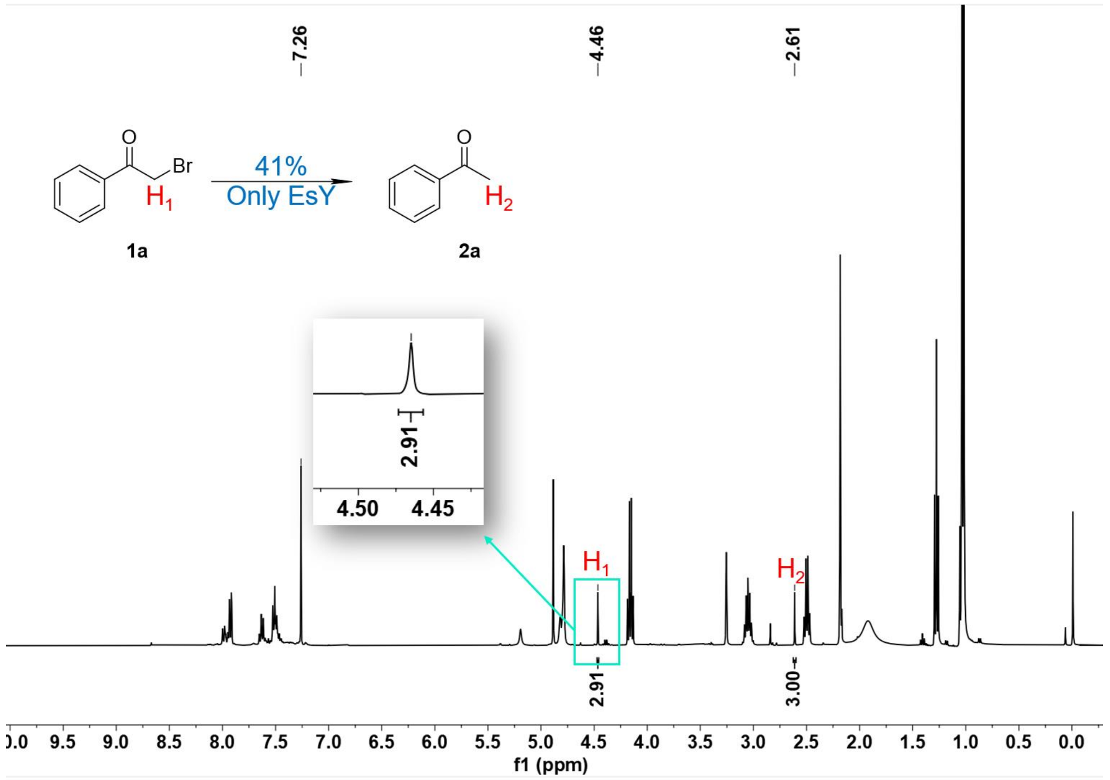


**Figure S22** $^1H$ NMR (400 MHz, $CDCl_3$, 298 K) spectrum of the reaction mixture of entry 2.

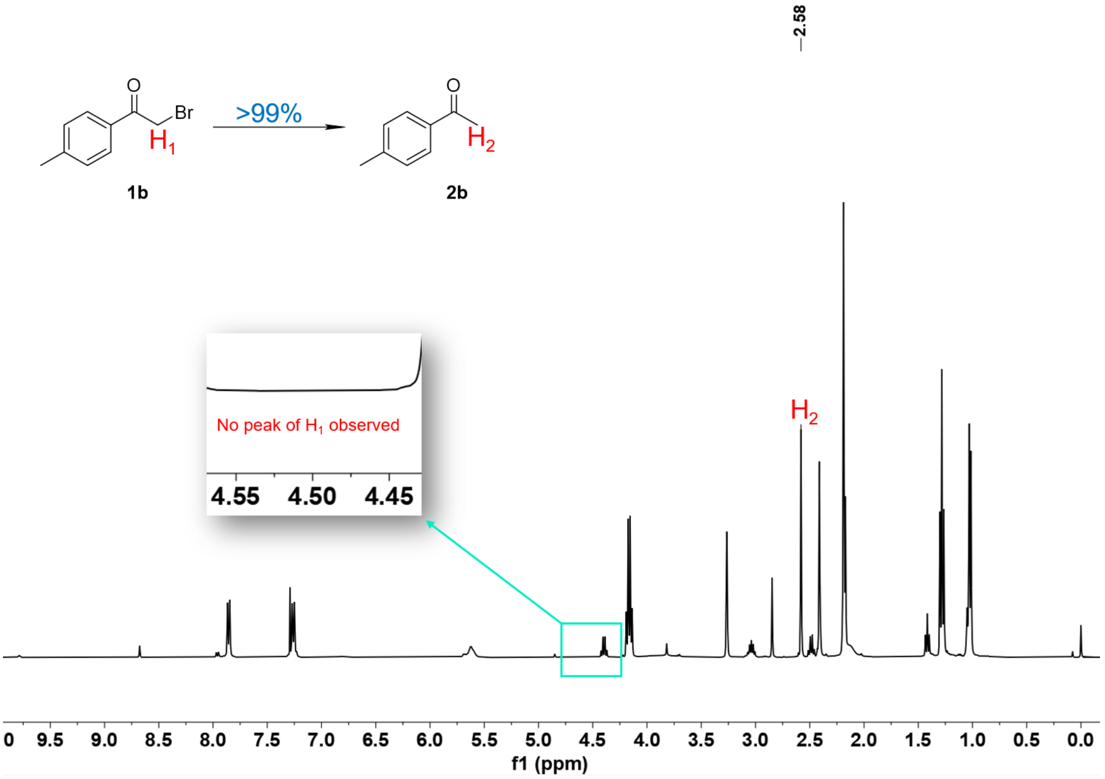


**Figure S23** $^1$H NMR (400 MHz, $CDCl_3$, 298 K) spectrum of the reaction mixture of entry 7.

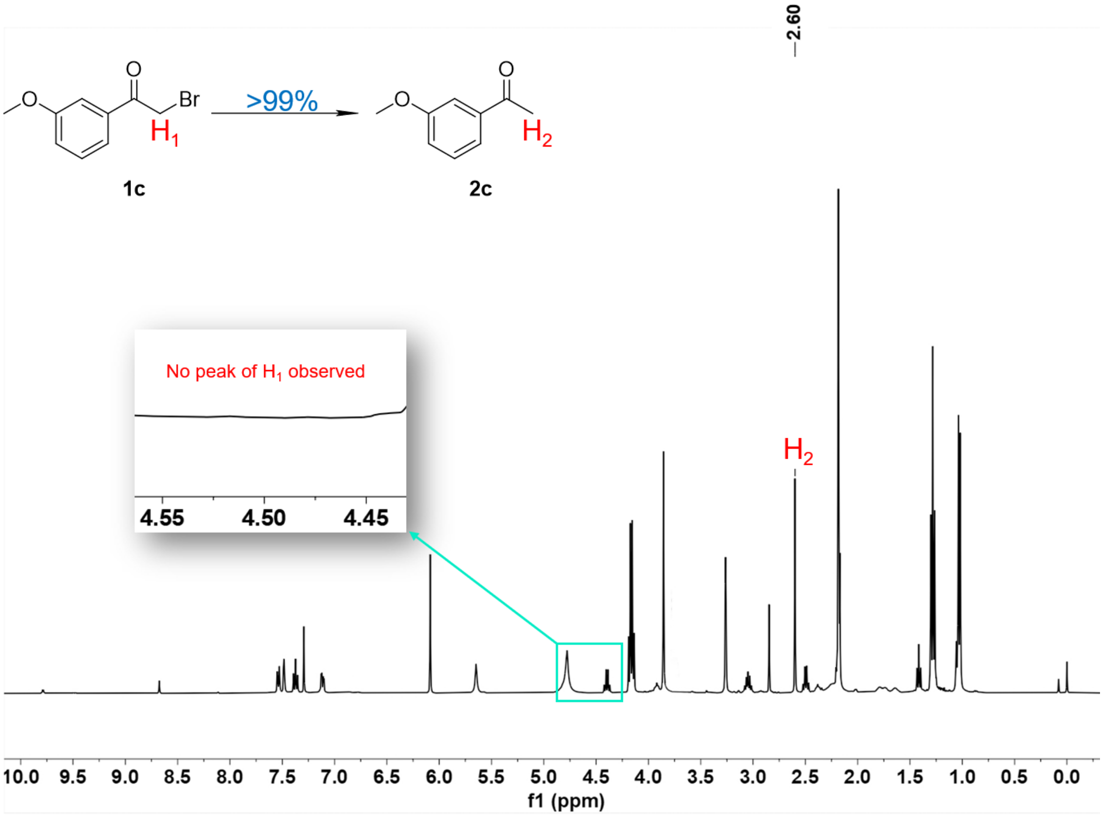


**Figure S24** $^1$H NMR (400 MHz, $CDCl_3$, 298 K) spectrum of the reaction mixture of entry 8.

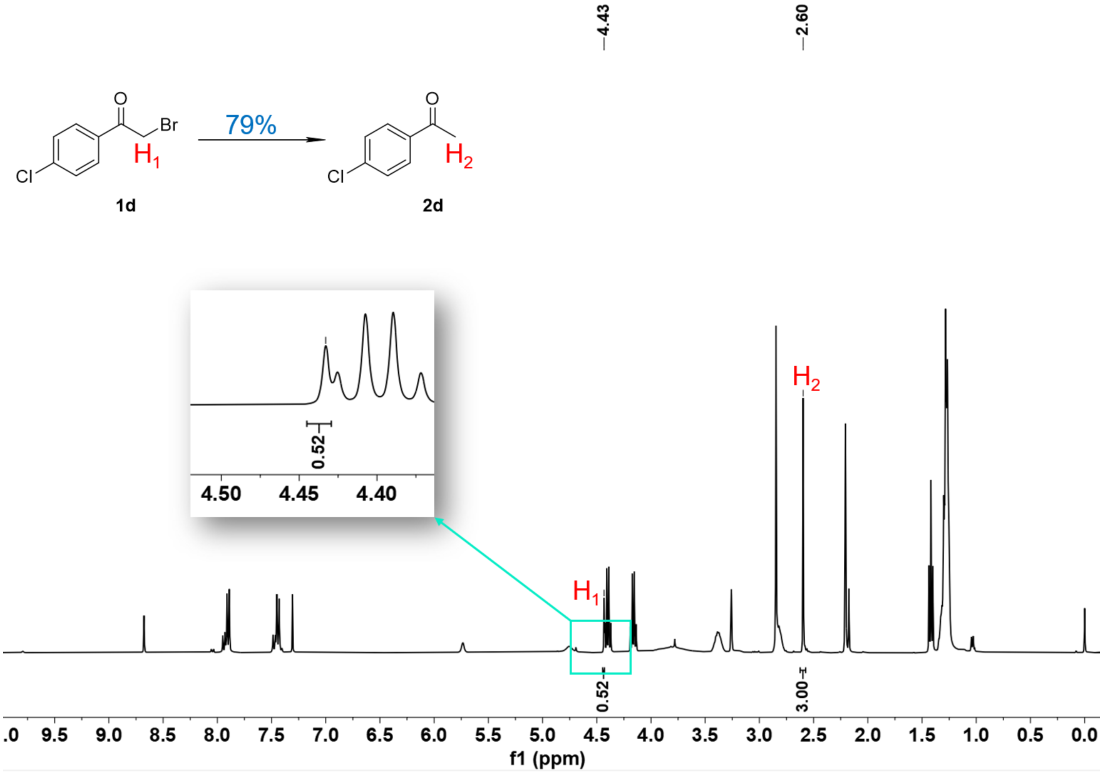


**Figure S25** $^{1}H$ NMR (400 MHz, $CDCl_3$, 298 K) spectrum of the reaction mixture of entry 9.

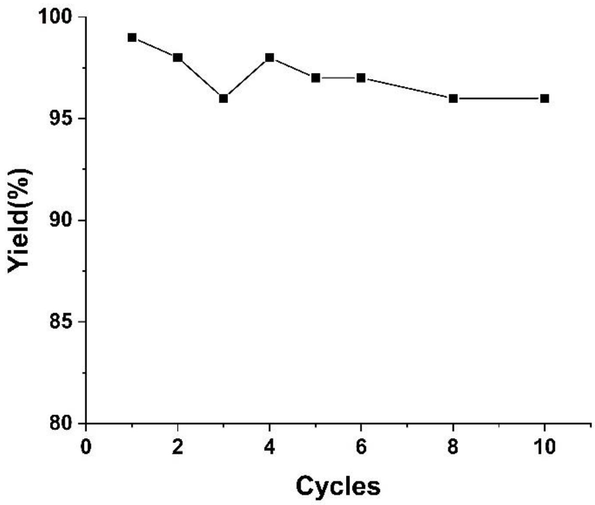


**Figure S26** Yields after different cycles of the reaction using catalyst ***m*-TPE Di-EtP5⊃G3**-EsY.

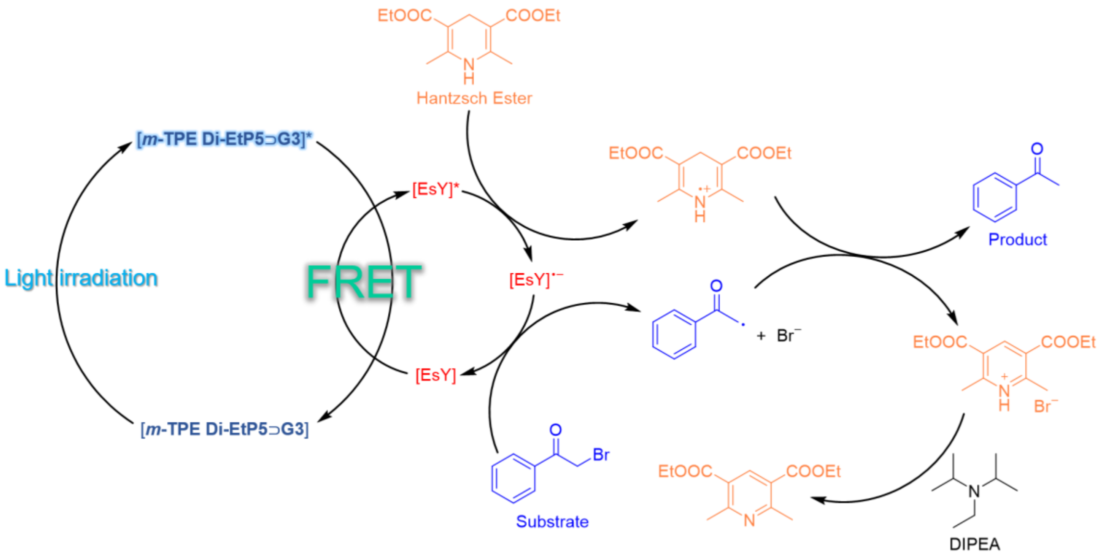


**Figure S27** Proposed mechanism for the 2-bromo-1-phenylethanone dehalogenation reaction mediated by ***m*-TPE Di-EtP5⊃G3**-EsY as a photocatalyst.[4]

## 6. X-ray crystallography data

**Table S2.** X-ray crystallography data.

| Structure name | *m*-TPE Di-EtP5 | *m*-TPE Di-EtP5⊃G1 | *m*-TPE Di-EtP5⊃G2 |
|---|---|---|---|
| **CCDC number** | 2154315 | 2174232 | 2194663 |
| **Formula sum** | C110 H136 O20 | C122 H152 N4 O20 | C130 H168 N4 O20 |
| **Formula weight** | 1776.96 g/mol | 1994.47 g/mol | 2106.67 g/mol |
| **Crystal system** | monoclinic | monoclinic | triclinic |
| **Space-group** | C 1 2/c 1 (15) | I 1 2/a 1 (15) | P -1 (2) |
| **Cell parameters** | a=45.5081(17) Å<br>b=12.6360(5) Å<br>c=22.2910(9) Å<br>β=116.231(2)° | a=22.6898(4) Å<br>b=12.6106(2) Å<br>c=40.8173(9) Å<br>β=92.842(1)° | a=12.6333(3) Å<br>b=20.5680(6) Å<br>c=22.9295(6) Å<br>α=86.143(1)°<br>β=88.731(1)°<br>γ=83.305(1)° |
| **Cell ratio** | a/b=3.6015<br>b/c=0.5669<br>c/a=0.4898 | a/b=1.7993<br>b/c=0.3090<br>c/a=1.7989 | a/b=0.6142<br>b/c=0.8970<br>c/a=1.8150 |
| **Cell volume** | 11498.2(8) $Å^3$ | 11664.77(40) $Å^3$ | 5903.39(30) $Å^3$ |
| **Z** | 230 | 4 | 2 |
| **Calc. density** | 1.12529 g/cm3 | 1.13562 $g/cm^3$ | 1.18508 $g/cm^3$ |
| **RAll** | 0.1346 | 0.0951 | 0.0955 |
| **Pearson code** | mC1144 | mI1256 | aP686 |
| **Formula type** | NO5P28Q35 | N2O10P61Q76 | N2O10P65Q84 |
| **Wyckoff sequence** | f143 | f157 | i343 |

## References


[1] T. Ogoshi, K. Kitajima, T. Aoki, S. Fujinami, T.-a. Yamagishi, Y. Nakamoto, *J. Org. Chem.* **2010**, *75*, 3268−3273.

[2] E. Meichsner, I. Nierengarten, M. Holler, M. Chessé, J.-F. Nierengarten, *Helv. Chim. Acta* **2018**, *101*, e1800059.

[3] M. H. Tootoonchi, G. Sharma, J. Calles, R. Prabhakar, A. E. Kaifer, *Angew. Chem.* **2016**, *128*, 11679−11683; *Angew. Chem. Int. Ed.*, 2016, **55**, 11507−11511.

[4] a) M. Neumann, S. Füldner, B. König, K. Zeitler, *Angew. Chem.* **2011**, *123*, 981−985; *Angew. Chem. Int. Ed.* **2011**, *50*, 951−954; b) Z. J. Wang, S. Ghasimi, K. Landfester, K. A. I. Zhang, *J. Mater. Chem. A* **2014**, *2*, 18720−18724; c) M. Hao, G. Sun, M. Zuo, Z. Xu, Y. Chen, X.-Y. Hu, L. Wang, *Angew. Chem.* **2020**, *132*, 10181−10186; *Angew. Chem. Int. Ed.* **2020**, *59*, 10095−10100; d) G. Sun, M. Zuo, W. Qian, J. Jiao, X.-Y. Hu, L. Wang, *Green Synth. & Catal.* **2021**, *2*, 32−37.
