# Supplementary material for: Dimeric Pillar[5]arene as a Novel Fluorescent Host for Controllable Fabrication of Supramolecular Assemblies and Their Photocatalytic Applications
Source: Adv Sci (Weinh). 2023 Jan 22;10(9):2206897. doi: 10.1002/advs.202206897 (PMC10037968; doi:10.1002/advs.202206897)

checkCIF/PLATON report

Structure factors have been supplied for datablock(s) 111

THIS REPORT IS FOR GUIDANCE ONLY. IF USED AS PART OF A REVIEW PROCEDURE FOR PUBLICATION, IT SHOULD NOT REPLACE THE EXPERTISE OF AN EXPERIENCED CRYSTALLOGRAPHIC REFEREE.

No syntax errors found. CIF dictionary Interpreting this report

Datablock: 111

Bond precision: C-C = 0.0032 A Wavelength=1.54178

Cell:	a=45.5081(17)	b=12.6360(5)	c=22.2910(9)
	alpha=90	beta=116.231(2)	gamma=90
Temperature:	150 K		

	Calculated	Reported
Volume	11498.2(8)	11498.2(8)
Space group	C 2/c	C 1 2/c 1
Hall group	-C 2yc	-C 2yc
Moiety formula	C110 H136 O20, 2(C H2 Cl2)	0.017(C110 H136 O20), 0.035(C H2 Cl2)
Sum formula	C112 H140 Cl4 O20	C1.95 H2.43 Cl0.07 O0.35
Mr	1948.04	33.88
Dx,g cm-3	1.125	1.125
Z	4	230
Mu (mm-1)	1.433	1.433
F000	4160.0	4160.0
F000'	4177.48	
h,k,lmax	56,15,27	56,15,27
Nref	11773	11652
Tmin,Tmax		0.011,0.087
Tmin'		

Correction method= # Reported T Limits: Tmin=0.011 Tmax=0.087
AbsCorr = NONE

Data completeness= 0.990 Theta(max)= 74.628

R(reflections)= 0.1193(8599) wR2(reflections)= 0.3513(11652)

S = 1.379 Npar= 566

The following ALERTS were generated. Each ALERT has the format
test-name_ALERT_alert-type_alert-level.
Click on the hyperlinks for more details of the test.

Alert level C

```
PLAT042_ALERT_1_C Calc. and Reported MoietyFormula Strings  Differ     Please Check
PLAT053_ALERT_1_C Minimum Crystal Dimension Missing (or Error) ...     Please Check
PLAT054_ALERT_1_C Medium  Crystal Dimension Missing (or Error) ...     Please Check
PLAT055_ALERT_1_C Maximum Crystal Dimension Missing (or Error) ...     Please Check
PLAT082_ALERT_2_C High R1 Value ..................................       0.12 Report
PLAT084_ALERT_3_C High wR2 Value (i.e. > 0.25) ...................       0.35 Report
PLAT094_ALERT_2_C Ratio of Maximum / Minimum Residual Density ....       2.92 Report
PLAT260_ALERT_2_C Large Average Ueq of Residue Including       Cl3      0.171 Check
PLAT260_ALERT_2_C Large Average Ueq of Residue Including       Cl1      0.172 Check
PLAT601_ALERT_2_C Unit Cell Contains Solvent Accessible VOIDS of .         73 Ang**3
PLAT906_ALERT_3_C Large K Value in the Analysis of Variance ......      5.980 Check
PLAT911_ALERT_3_C Missing FCF Refl Between Thmin & STh/L=    0.600         31 Report
PLAT918_ALERT_3_C Reflection(s) with I(obs) much Smaller I(calc) .          2 Check
PLAT939_ALERT_3_C Large Value of Not (SHELXL) Weight Optimized S .      12.54 Check
```

Alert level G

```
FORMU01_ALERT_1_G  There is a discrepancy between the atom counts in the
            _chemical_formula_sum and _chemical_formula_moiety. This is
            usually due to the moiety formula being in the wrong format.
            Atom count from _chemical_formula_sum:   C1.95 H2.43 Cl.07 O0.35
            Atom count from _chemical_formula_moiety:C1.905 H2.382 Cl.07 O0.34
CELLZ01_ALERT_1_G Difference between formula and atom_site contents detected.
CELLZ01_ALERT_1_G ALERT: Large difference may be due to a
            symmetry error - see SYMMG tests
           From the CIF: _cell_formula_units_Z  230
           From the CIF: _chemical_formula_sum  C1.95 H2.43 Cl0.07 O0.35
           TEST: Compare cell contents of formula and atom_site data

           atom    Z*formula  cif sites diff
           C        448.50    448.00    0.50
           H        558.90    560.00   -1.10
           Cl        16.10     16.00    0.10
           O         80.50     80.00    0.50
PLAT002_ALERT_2_G Number of Distance or Angle Restraints on AtSite         46 Note
PLAT045_ALERT_1_G Calculated and Reported Z Differ by a Factor ...      0.017 Check
PLAT072_ALERT_2_G SHELXL First  Parameter in WGHT  Unusually Large       0.20 Report
PLAT128_ALERT_4_G Alternate Setting for Input Space Group     C2/c       I2/a Note
PLAT171_ALERT_4_G The CIF-Embedded .res File Contains EADP Records          2 Report
PLAT176_ALERT_4_G The CIF-Embedded .res File Contains SADI Records          4 Report
PLAT300_ALERT_4_G Atom Site Occupancy of Cl3        Constrained at        0.6 Check
PLAT300_ALERT_4_G Atom Site Occupancy of Cl4        Constrained at        0.6 Check
PLAT300_ALERT_4_G Atom Site Occupancy of C6         Constrained at        0.6 Check
PLAT300_ALERT_4_G Atom Site Occupancy of H6A        Constrained at        0.6 Check
PLAT300_ALERT_4_G Atom Site Occupancy of H6B        Constrained at        0.6 Check
PLAT300_ALERT_4_G Atom Site Occupancy of Cl1        Constrained at        0.4 Check
PLAT300_ALERT_4_G Atom Site Occupancy of Cl2        Constrained at        0.4 Check
PLAT300_ALERT_4_G Atom Site Occupancy of C3         Constrained at        0.4 Check
PLAT300_ALERT_4_G Atom Site Occupancy of H3A        Constrained at        0.4 Check
PLAT300_ALERT_4_G Atom Site Occupancy of H3B        Constrained at        0.4 Check
```

```
PLAT302_ALERT_4_G Anion/Solvent/Minor-Residue Disorder (Resd  2  )       100% Note
PLAT302_ALERT_4_G Anion/Solvent/Minor-Residue Disorder (Resd  3  )       100% Note
PLAT432_ALERT_2_G Short Inter X...Y Contact  Cl1      ..C36      .       3.25 Ang.
                                                     x,y,z  =      1_555 Check
PLAT860_ALERT_3_G Number of Least-Squares Restraints ............         92 Note
PLAT912_ALERT_4_G Missing # of FCF Reflections Above STh/L=  0.600       90 Note
PLAT913_ALERT_3_G Missing # of Very Strong Reflections in FCF ....         2 Note
PLAT933_ALERT_2_G Number of HKL-OMIT Records in Embedded .res File         8 Note
PLAT941_ALERT_3_G Average HKL Measurement Multiplicity ...........       3.1 Low
PLAT978_ALERT_2_G Number C-C Bonds with Positive Residual Density.         1 Info
PLAT992_ALERT_5_G Repd & Actual _reflns_number_gt Values Differ by         2 Check
```

```
  0 ALERT level A = Most likely a serious problem - resolve or explain
  0 ALERT level B = A potentially serious problem, consider carefully
 14 ALERT level C = Check. Ensure it is not caused by an omission or oversight
 29 ALERT level G = General information/check it is not something unexpected

  8 ALERT type 1 CIF construction/syntax error, inconsistent or missing data
 10 ALERT type 2 Indicator that the structure model may be wrong or deficient
  8 ALERT type 3 Indicator that the structure quality may be low
 16 ALERT type 4 Improvement, methodology, query or suggestion
  1 ALERT type 5 Informative message, check
```

It is advisable to attempt to resolve as many as possible of the alerts in all categories. Often the minor alerts point to easily fixed oversights, errors and omissions in your CIF or refinement strategy, so attention to these fine details can be worthwhile. In order to resolve some of the more serious problems it may be necessary to carry out additional measurements or structure refinements. However, the purpose of your study may justify the reported deviations and the more serious of these should normally be commented upon in the discussion or experimental section of a paper or in the "special_details" fields of the CIF. checkCIF was carefully designed to identify outliers and unusual parameters, but every test has its limitations and alerts that are not important in a particular case may appear. Conversely, the absence of alerts does not guarantee there are no aspects of the results needing attention. It is up to the individual to critically assess their own results and, if necessary, seek expert advice.

Publication of your CIF in IUCr journals

A basic structural check has been run on your CIF. These basic checks will be run on all CIFs submitted for publication in IUCr journals (*Acta Crystallographica*, *Journal of Applied Crystallography*, *Journal of Synchrotron Radiation*); however, if you intend to submit to *Acta Crystallographica Section C* or *E* or *IUCrData*, you should make sure that full publication checks are run on the final version of your CIF prior to submission.

Publication of your CIF in other journals

Please refer to the *Notes for Authors* of the relevant journal for any special instructions relating to CIF submission.

PLATON version of 09/05/2022; check.def file version of 21/03/2022

Datablock 111 - ellipsoid plot

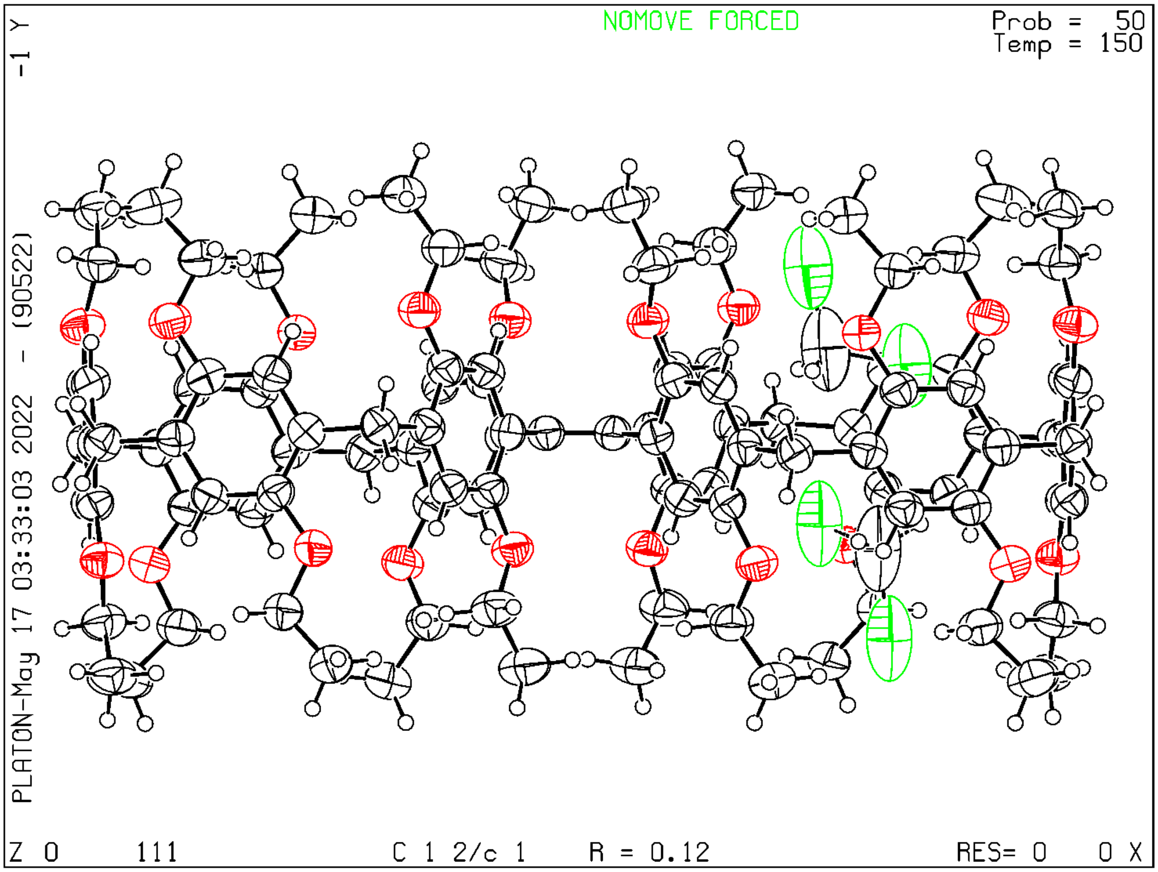

Supplement: Supplementary file 2 — Supporting Information [file ADVS-10-2206897-s002.zip › EtP5 Dimer.pdf]
