# Supplementary material for: Dimeric Pillar[5]arene as a Novel Fluorescent Host for Controllable Fabrication of Supramolecular Assemblies and Their Photocatalytic Applications
Source: Adv Sci (Weinh). 2023 Jan 22;10(9):2206897. doi: 10.1002/advs.202206897 (PMC10037968; doi:10.1002/advs.202206897)

checkCIF/PLATON report

You have not supplied any structure factors. As a result the full set of tests cannot be run.

THIS REPORT IS FOR GUIDANCE ONLY. IF USED AS PART OF A REVIEW PROCEDURE FOR PUBLICATION, IT SHOULD NOT REPLACE THE EXPERTISE OF AN EXPERIENCED CRYSTALLOGRAPHIC REFEREE.

No syntax errors found. CIF dictionary Interpreting this report

Datablock: 111_sq

Bond precision:	C-C = 0.0041 A		Wavelength=1.54178
Cell:	a=22.6898(4)	b=12.6106(2)	c=40.8173(9)
	alpha=90	beta=92.842(1)	gamma=90
Temperature:	193 K		

	Calculated	Reported
Volume	11664.8(4)	11664.8(4)
Space group	I 2/a	I 1 2/a 1
Hall group	-I 2ya	-I 2ya
Moiety formula	C110 H136 O20, 2(C6 H8 N2) [+ solvent]	C110 H136 O20, 2(C6 H8 N2)
Sum formula	C122 H152 N4 O20 [+ solvent]	C122 H152 N4 O20
Mr	1994.48	1994.47
Dx,g cm-3	1.136	1.136
Z	4	4
Mu (mm-1)	0.612	0.612
F000	4288.0	4288.0
F000'	4300.72	
h,k,lmax	27,15,48	27,15,48
Nref	10297	10288
Tmin,Tmax		
Tmin'		

Correction method= Not given

Data completeness= 0.999 Theta(max)= 66.592

R(reflections)= 0.0773(7777) wR2(reflections)= 0.2471(10288)

S = 1.048 Npar= 705

The following ALERTS were generated. Each ALERT has the format
test-name_ALERT_alert-type_alert-level.
Click on the hyperlinks for more details of the test.

Alert level C

```
DIFMN02_ALERT_2_C  The minimum difference density is < -0.1*ZMAX*0.75
            _refine_diff_density_min given =      -0.625
            Test value =      -0.600
DIFMN03_ALERT_1_C  The minimum difference density is < -0.1*ZMAX*0.75
            The relevant atom site should be identified.
DIFMX02_ALERT_1_C  The maximum difference density is > 0.1*ZMAX*0.75
            The relevant atom site should be identified.
PLAT053_ALERT_1_C Minimum Crystal Dimension Missing (or Error) ...     Please Check
PLAT054_ALERT_1_C Medium  Crystal Dimension Missing (or Error) ...     Please Check
PLAT055_ALERT_1_C Maximum Crystal Dimension Missing (or Error) ...     Please Check
PLAT097_ALERT_2_C Large Reported Max.  (Positive) Residual Density       0.80 eA-3
PLAT098_ALERT_2_C Large Reported Min.  (Negative) Residual Density      -0.62 eA-3
PLAT234_ALERT_4_C Large Hirshfeld Difference N11      --C6       .       0.20 Ang.
PLAT234_ALERT_4_C Large Hirshfeld Difference C2       --C3       .       0.16 Ang.
PLAT250_ALERT_2_C Large U3/U1 Ratio for Average U(i,j) Tensor ....        3.2 Note
PLAT260_ALERT_2_C Large Average Ueq of Residue Including       N10      0.206 Check
PLAT329_ALERT_4_C Carbon Atom Hybridisation Unclear for .........          C5 Check
PLAT340_ALERT_3_C Low Bond Precision on  C-C Bonds ..............     0.00413 Ang.
```

Alert level G

```
PLAT002_ALERT_2_G Number of Distance or Angle Restraints on AtSite         12 Note
PLAT003_ALERT_2_G Number of Uiso or Uij Restrained non-H Atoms ...         12 Report
PLAT012_ALERT_1_G No       _shelx_res_checksum Found in CIF ......     Please Check
PLAT014_ALERT_1_G No       _shelx_fab_checksum Found in CIF ......     Please Check
PLAT072_ALERT_2_G SHELXL First  Parameter in WGHT  Unusually Large       0.15 Report
PLAT083_ALERT_2_G SHELXL Second Parameter in WGHT  Unusually Large      12.87 Why ?
PLAT172_ALERT_4_G The CIF-Embedded .res File Contains DFIX Records          5 Report
PLAT173_ALERT_4_G The CIF-Embedded .res File Contains DANG Records          4 Report
PLAT176_ALERT_4_G The CIF-Embedded .res File Contains SADI Records          1 Report
PLAT178_ALERT_4_G The CIF-Embedded .res File Contains SIMU Records          1 Report
PLAT231_ALERT_4_G Hirshfeld Test (Solvent)   N10      --C5       .        5.3 s.u.
PLAT302_ALERT_4_G Anion/Solvent/Minor-Residue Disorder (Resd  2  )        50% Note
PLAT367_ALERT_2_G Long?  C(sp?)-C(sp?) Bond  C2       - C3       .       1.52 Ang.
PLAT605_ALERT_4_G Largest Solvent Accessible VOID in the Structure         10 A**3
PLAT720_ALERT_4_G Number of Unusual/Non-Standard Labels .........         123 Note
PLAT860_ALERT_3_G Number of Least-Squares Restraints ............         130 Note
PLAT869_ALERT_4_G ALERTS Related to the Use of SQUEEZE  Suppressed          ! Info
PLAT933_ALERT_2_G Number of HKL-OMIT Records in Embedded .res File          8 Note
```

0 **ALERT level A** = Most likely a serious problem - resolve or explain
0 **ALERT level B** = A potentially serious problem, consider carefully
14 **ALERT level C** = Check. Ensure it is not caused by an omission or oversight
18 **ALERT level G** = General information/check it is not something unexpected

7 ALERT type 1 CIF construction/syntax error, inconsistent or missing data

```
 11 ALERT type 2 Indicator that the structure model may be wrong or deficient
  2 ALERT type 3 Indicator that the structure quality may be low
 12 ALERT type 4 Improvement, methodology, query or suggestion
  0 ALERT type 5 Informative message, check
```

Validation response form

Please find below a validation response form (VRF) that can be filled in and pasted into your CIF.

```
# start Validation Reply Form
_vrf_DIFMN02_111_sq
;
PROBLEM: The minimum difference density is < -0.1*ZMAX*0.75
RESPONSE: ...
;
_vrf_DIFMN03_111_sq
;
PROBLEM: The minimum difference density is < -0.1*ZMAX*0.75
RESPONSE: ...
;
_vrf_DIFMX02_111_sq
;
PROBLEM: The maximum difference density is > 0.1*ZMAX*0.75
RESPONSE: ...
;
_vrf_PLAT053_111_sq
;
PROBLEM: Minimum Crystal Dimension Missing (or Error) ...     Please Check
RESPONSE: ...
;
_vrf_PLAT054_111_sq
;
PROBLEM: Medium  Crystal Dimension Missing (or Error) ...     Please Check
RESPONSE: ...
;
_vrf_PLAT055_111_sq
;
PROBLEM: Maximum Crystal Dimension Missing (or Error) ...     Please Check
RESPONSE: ...
;
_vrf_PLAT097_111_sq
;
PROBLEM: Large Reported Max.  (Positive) Residual Density       0.80 eA-3
RESPONSE: ...
;
_vrf_PLAT098_111_sq
;
PROBLEM: Large Reported Min.  (Negative) Residual Density      -0.62 eA-3
RESPONSE: ...
;
_vrf_PLAT234_111_sq
;
PROBLEM: Large Hirshfeld Difference N11      --C6       .       0.20 Ang.
RESPONSE: ...
;
```

```
_vrf_PLAT250_111_sq
;
PROBLEM: Large U3/U1 Ratio for Average U(i,j) Tensor ....        3.2 Note
RESPONSE: ...
;
_vrf_PLAT260_111_sq
;
PROBLEM: Large Average Ueq of Residue Including       N10      0.206 Check
RESPONSE: ...
;
_vrf_PLAT329_111_sq
;
PROBLEM: Carbon Atom Hybridisation Unclear for .........         C5 Check
RESPONSE: ...
;
_vrf_PLAT340_111_sq
;
PROBLEM: Low Bond Precision on  C-C Bonds ..............    0.00413 Ang.
RESPONSE: ...
;
# end Validation Reply Form
```

It is advisable to attempt to resolve as many as possible of the alerts in all categories. Often the minor alerts point to easily fixed oversights, errors and omissions in your CIF or refinement strategy, so attention to these fine details can be worthwhile. In order to resolve some of the more serious problems it may be necessary to carry out additional measurements or structure refinements. However, the purpose of your study may justify the reported deviations and the more serious of these should normally be commented upon in the discussion or experimental section of a paper or in the "special_details" fields of the CIF. checkCIF was carefully designed to identify outliers and unusual parameters, but every test has its limitations and alerts that are not important in a particular case may appear. Conversely, the absence of alerts does not guarantee there are no aspects of the results needing attention. It is up to the individual to critically assess their own results and, if necessary, seek expert advice.

Publication of your CIF in IUCr journals

A basic structural check has been run on your CIF. These basic checks will be run on all CIFs submitted for publication in IUCr journals (*Acta Crystallographica*, *Journal of Applied Crystallography*, *Journal of Synchrotron Radiation*); however, if you intend to submit to *Acta Crystallographica Section C* or *E* or *IUCrData*, you should make sure that full publication checks are run on the final version of your CIF prior to submission.

Publication of your CIF in other journals

Please refer to the *Notes for Authors* of the relevant journal for any special instructions relating to CIF submission.

PLATON version of 09/05/2022; check.def file version of 21/03/2022

Datablock 111_sq - ellipsoid plot

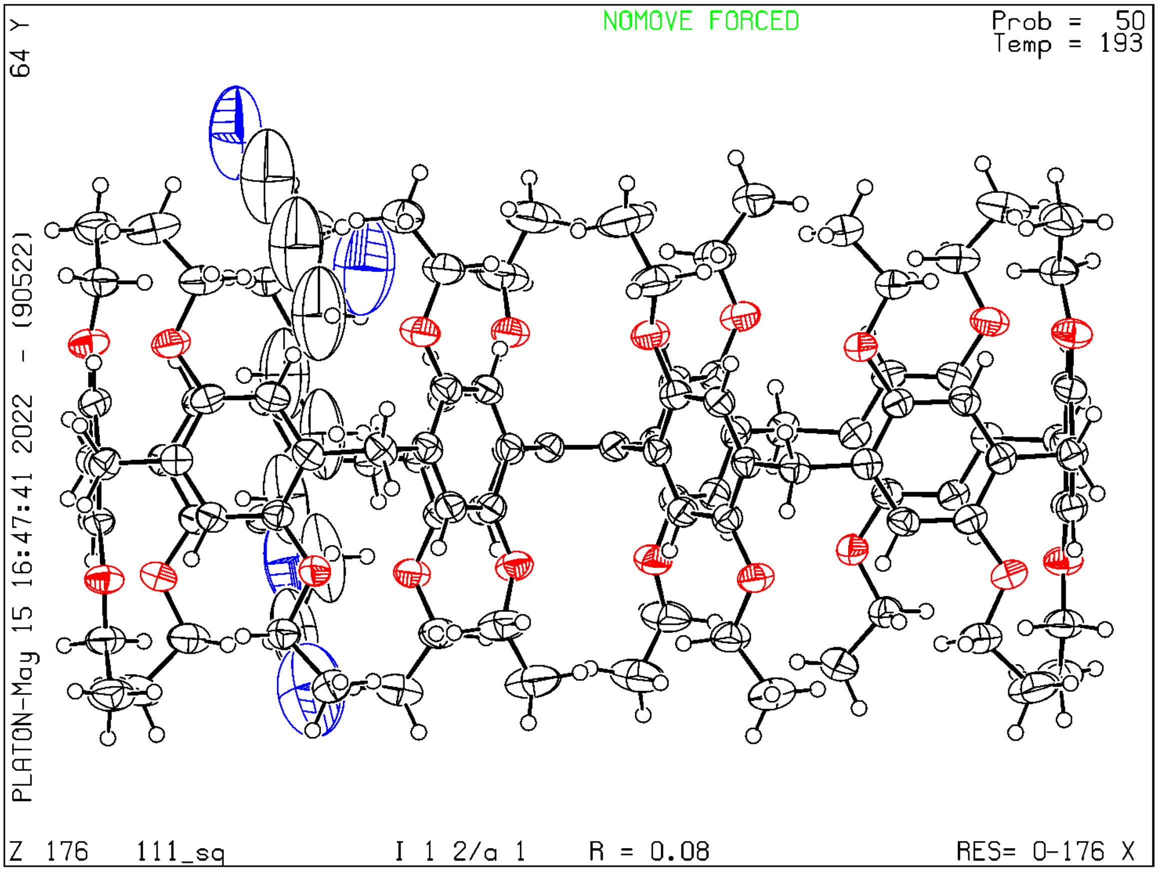

Supplement: Supplementary file 2 — Supporting Information [file ADVS-10-2206897-s002.zip › EtP5 Dimer-G1.pdf]
