# Supplementary material for: Dimeric Pillar[5]arene as a Novel Fluorescent Host for Controllable Fabrication of Supramolecular Assemblies and Their Photocatalytic Applications
Source: Adv Sci (Weinh). 2023 Jan 22;10(9):2206897. doi: 10.1002/advs.202206897 (PMC10037968; doi:10.1002/advs.202206897)

checkCIF/PLATON report

Structure factors have been supplied for datablock(s) wky

THIS REPORT IS FOR GUIDANCE ONLY. IF USED AS PART OF A REVIEW PROCEDURE FOR PUBLICATION, IT SHOULD NOT REPLACE THE EXPERTISE OF AN EXPERIENCED CRYSTALLOGRAPHIC REFEREE.

No syntax errors found. CIF dictionary Interpreting this report

Datablock: wky

```
Bond precision:   C-C = 0.0041 A          Wavelength=1.54178

Cell:            a=12.6333(3)       b=20.5680(6)      c=22.9295(6)
                 alpha=86.143(1)    beta=88.731(1)    gamma=83.305(1)
Temperature:     150 K
```

	Calculated	Reported
Volume	5903.4(3)	5903.4(3)
Space group	P -1	P -1
Hall group	-P 1	-P 1
Moiety formula	C110 H136 O20, 2(C10 H16 N2) [+ solvent]	C110 H136 O20, 2(C10 H16 N2)
Sum formula	C130 H168 N4 O20 [+ solvent]	C130 H168 N4 O20
Mr	2106.69	2106.67
Dx,g cm-3	1.185	1.185
Z	2	2
Mu (mm-1)	0.629	0.629
F000	2272.0	2272.0
F000'	2278.63	
h,k,lmax	15,24,27	15,24,27
Nref	20844	20804
Tmin,Tmax	0.871,0.927	0.011,0.087
Tmin'	0.871	

```
Correction method= # Reported T Limits: Tmin=0.011 Tmax=0.087
AbsCorr = MULTI-SCAN

Data completeness= 0.998            Theta(max)= 66.595
```

R(reflections)= 0.0808(15922) wR2(reflections)= 0.2590(20804)

S = 1.045 Npar= 1462

The following ALERTS were generated. Each ALERT has the format
test-name_ALERT_alert-type_alert-level.
Click on the hyperlinks for more details of the test.

Alert level C

```
DIFMN02_ALERT_2_C  The minimum difference density is < -0.1*ZMAX*0.75
            _refine_diff_density_min given =     -0.604
            Test value =     -0.600
DIFMN03_ALERT_1_C  The minimum difference density is < -0.1*ZMAX*0.75
            The relevant atom site should be identified.
DIFMX02_ALERT_1_C  The maximum difference density is > 0.1*ZMAX*0.75
            The relevant atom site should be identified.
PLAT084_ALERT_3_C High wR2 Value (i.e. > 0.25) ..................       0.26 Report
PLAT097_ALERT_2_C Large Reported Max.  (Positive) Residual Density       0.72 eA-3
PLAT220_ALERT_2_C NonSolvent   Resd 1  C   Ueq(max)/Ueq(min) Range        3.1 Ratio
PLAT241_ALERT_2_C High   'MainMol' Ueq as Compared to Neighbors of       C042 Check
PLAT241_ALERT_2_C High   'MainMol' Ueq as Compared to Neighbors of         C1 Check
PLAT241_ALERT_2_C High   'MainMol' Ueq as Compared to Neighbors of       C03T Check
PLAT242_ALERT_2_C Low    'MainMol' Ueq as Compared to Neighbors of       C03L Check
PLAT242_ALERT_2_C Low    'MainMol' Ueq as Compared to Neighbors of       C03S Check
PLAT260_ALERT_2_C Large Average Ueq of Residue Including        N1      0.153 Check
PLAT260_ALERT_2_C Large Average Ueq of Residue Including      N03Q      0.128 Check
PLAT329_ALERT_4_C Carbon Atom Hybridisation Unclear for .........       C03T Check
PLAT340_ALERT_3_C Low Bond Precision on  C-C Bonds ...............    0.00406 Ang.
PLAT360_ALERT_2_C Short  C(sp3)-C(sp3) Bond  C042     - C043     .       1.40 Ang.
PLAT360_ALERT_2_C Short  C(sp3)-C(sp3) Bond  C03X     - C041     .       1.42 Ang.
PLAT906_ALERT_3_C Large K Value in the Analysis of Variance ......      2.499 Check
PLAT911_ALERT_3_C Missing FCF Refl Between Thmin & STh/L=    0.595         42 Report
PLAT918_ALERT_3_C Reflection(s) with I(obs) much Smaller I(calc) .          3 Check
PLAT977_ALERT_2_C Check Negative Difference Density on H1AA      .      -0.37 eA-3
PLAT977_ALERT_2_C Check Negative Difference Density on H1BC      .      -0.36 eA-3
PLAT992_ALERT_5_C Repd & Actual _reflns_number_gt Values Differ by         25 Check
```

Alert level G

```
PLAT002_ALERT_2_G Number of Distance or Angle Restraints on AtSite         17 Note
PLAT003_ALERT_2_G Number of Uiso or Uij Restrained non-H Atoms ...        160 Report
PLAT072_ALERT_2_G SHELXL First  Parameter in WGHT  Unusually Large       0.17 Report
PLAT154_ALERT_1_G The s.u.'s on the Cell Angles are Equal ..(Note)      0.001 Degree
PLAT172_ALERT_4_G The CIF-Embedded .res File Contains DFIX Records          2 Report
PLAT178_ALERT_4_G The CIF-Embedded .res File Contains SIMU Records          2 Report
PLAT187_ALERT_4_G The CIF-Embedded .res File Contains RIGU Records          2 Report
PLAT300_ALERT_4_G Atom Site Occupancy of C10       Constrained at         0.5 Check
PLAT300_ALERT_4_G Atom Site Occupancy of C11       Constrained at         0.5 Check
PLAT300_ALERT_4_G Atom Site Occupancy of C03P      Constrained at         0.5 Check
PLAT300_ALERT_4_G Atom Site Occupancy of C045      Constrained at         0.5 Check
PLAT300_ALERT_4_G Atom Site Occupancy of H10A      Constrained at         0.5 Check
PLAT300_ALERT_4_G Atom Site Occupancy of H10B      Constrained at         0.5 Check
PLAT300_ALERT_4_G Atom Site Occupancy of H11A      Constrained at         0.5 Check
PLAT300_ALERT_4_G Atom Site Occupancy of H11B      Constrained at         0.5 Check
```

```
PLAT300_ALERT_4_G Atom Site Occupancy of H11C       Constrained at        0.5 Check
PLAT300_ALERT_4_G Atom Site Occupancy of H20        Constrained at        0.5 Check
PLAT300_ALERT_4_G Atom Site Occupancy of H42        Constrained at        0.5 Check
PLAT300_ALERT_4_G Atom Site Occupancy of H04A       Constrained at        0.5 Check
PLAT300_ALERT_4_G Atom Site Occupancy of H04B       Constrained at        0.5 Check
PLAT300_ALERT_4_G Atom Site Occupancy of H04C       Constrained at        0.5 Check
PLAT300_ALERT_4_G Atom Site Occupancy of N1         Constrained at        0.5 Check
PLAT300_ALERT_4_G Atom Site Occupancy of N6         Constrained at        0.5 Check
PLAT300_ALERT_4_G Atom Site Occupancy of C4         Constrained at        0.5 Check
PLAT300_ALERT_4_G Atom Site Occupancy of C5         Constrained at        0.5 Check
PLAT300_ALERT_4_G Atom Site Occupancy of C7         Constrained at        0.5 Check
PLAT300_ALERT_4_G Atom Site Occupancy of C9         Constrained at        0.5 Check
PLAT300_ALERT_4_G Atom Site Occupancy of H4A        Constrained at        0.5 Check
PLAT300_ALERT_4_G Atom Site Occupancy of H4B        Constrained at        0.5 Check
PLAT300_ALERT_4_G Atom Site Occupancy of H5A        Constrained at        0.5 Check
PLAT300_ALERT_4_G Atom Site Occupancy of H5B        Constrained at        0.5 Check
PLAT300_ALERT_4_G Atom Site Occupancy of H2AA       Constrained at        0.5 Check
PLAT300_ALERT_4_G Atom Site Occupancy of H2AB       Constrained at        0.5 Check
PLAT300_ALERT_4_G Atom Site Occupancy of H2BC       Constrained at        0.5 Check
PLAT300_ALERT_4_G Atom Site Occupancy of H2BD       Constrained at        0.5 Check
PLAT300_ALERT_4_G Atom Site Occupancy of C03Y       Constrained at       0.75 Check
PLAT300_ALERT_4_G Atom Site Occupancy of C6         Constrained at       0.25 Check
PLAT300_ALERT_4_G Atom Site Occupancy of H24        Constrained at        0.5 Check
PLAT300_ALERT_4_G Atom Site Occupancy of H26        Constrained at       0.75 Check
PLAT300_ALERT_4_G Atom Site Occupancy of H45        Constrained at        0.5 Check
PLAT300_ALERT_4_G Atom Site Occupancy of H46        Constrained at        0.5 Check
PLAT300_ALERT_4_G Atom Site Occupancy of H47        Constrained at        0.5 Check
PLAT300_ALERT_4_G Atom Site Occupancy of H50        Constrained at       0.75 Check
PLAT300_ALERT_4_G Atom Site Occupancy of H1AA       Constrained at        0.5 Check
PLAT300_ALERT_4_G Atom Site Occupancy of H1AB       Constrained at        0.5 Check
PLAT300_ALERT_4_G Atom Site Occupancy of H1BC       Constrained at        0.5 Check
PLAT300_ALERT_4_G Atom Site Occupancy of H1BD       Constrained at        0.5 Check
PLAT300_ALERT_4_G Atom Site Occupancy of H6A        Constrained at       0.25 Check
PLAT300_ALERT_4_G Atom Site Occupancy of H6B        Constrained at       0.25 Check
PLAT301_ALERT_3_G Main Residue  Disorder .............(Resd  1  )          2% Note
PLAT302_ALERT_4_G Anion/Solvent/Minor-Residue Disorder (Resd  2  )         25% Note
PLAT302_ALERT_4_G Anion/Solvent/Minor-Residue Disorder (Resd  3  )          8% Note
PLAT343_ALERT_2_G Unusual sp?      Angle Range in Main Residue for         C8 Check
PLAT367_ALERT_2_G Long?  C(sp?)-C(sp?) Bond  C1       - C8       .       1.50 Ang.
PLAT367_ALERT_2_G Long?  C(sp?)-C(sp?) Bond  C03K     - C03T     .       1.50 Ang.
PLAT410_ALERT_2_G Short Intra H...H Contact  H10B     ..H026     .       2.07 Ang.
                                                        x,y,z  =     1_555 Check
PLAT410_ALERT_2_G Short Intra H...H Contact  H35      ..H47      .       2.11 Ang.
                                                        x,y,z  =     1_555 Check
PLAT411_ALERT_2_G Short Inter H...H Contact  H03T     ..H6B      .       1.92 Ang.
                                                        x,y,z  =     1_555 Check
PLAT411_ALERT_2_G Short Inter H...H Contact  H5A      ..Hm       .       2.01 Ang.
                                                        x,y,z  =     1_555 Check
PLAT412_ALERT_2_G Short Intra XH3 .. XHn     H11A     ..H03S     .       2.10 Ang.
                                                        x,y,z  =     1_555 Check
PLAT432_ALERT_2_G Short Inter X...Y Contact  N1       ..C043     .       2.99 Ang.
                                                      1+x,y,z  =     1_655 Check
PLAT432_ALERT_2_G Short Inter X...Y Contact  N044     ..C4       .       2.93 Ang.
                                                     -1+x,y,z  =     1_455 Check
PLAT432_ALERT_2_G Short Inter X...Y Contact  C3       ..C9       .       3.03 Ang.
                                                     -1+x,y,z  =     1_455 Check
PLAT605_ALERT_4_G Largest Solvent Accessible VOID in the Structure         10 A**3
```

```
PLAT720_ALERT_4_G Number of Unusual/Non-Standard Labels ..........        268 Note
PLAT773_ALERT_2_G Check long C-C Bond in CIF: C03T     --C03Y            1.79 Ang.
PLAT773_ALERT_2_G Check long C-C Bond in CIF: C045     --C10             1.93 Ang.
PLAT773_ALERT_2_G Check long C-C Bond in CIF: C4       --C7              2.03 Ang.
PLAT779_ALERT_4_G Suspect or Irrelevant (Bond) Angle(s) in CIF ...      31.20 Deg.
              C03P -O00I -C10     1_555   1_555   1_555 ......     #   20 Check
PLAT779_ALERT_4_G Suspect or Irrelevant (Bond) Angle(s) in CIF ...      30.80 Deg.
              C10  -C03P -H20     1_555   1_555   1_555 ......     #  516 Check
PLAT779_ALERT_4_G Suspect or Irrelevant (Bond) Angle(s) in CIF ...      34.80 Deg.
              H24  -C03T -H47     1_555   1_555   1_555 ......     #  538 Check
PLAT779_ALERT_4_G Suspect or Irrelevant (Bond) Angle(s) in CIF ...      22.20 Deg.
              C03P -C045 -C10     1_555   1_555   1_555 ......     #  596 Check
PLAT779_ALERT_4_G Suspect or Irrelevant (Bond) Angle(s) in CIF ...      34.60 Deg.
              H1AA -C1   -H1BC    1_555   1_555   1_555 ......     #  609 Check
PLAT779_ALERT_4_G Suspect or Irrelevant (Bond) Angle(s) in CIF ...      32.90 Deg.
              H2AA -C2   -H2BD    1_555   1_555   1_555 ......     #  632 Check
PLAT779_ALERT_4_G Suspect or Irrelevant (Bond) Angle(s) in CIF ...      35.90 Deg.
              C4   -C2   -H2BC    1_555   1_555   1_555 ......     #  639 Check
PLAT779_ALERT_4_G Suspect or Irrelevant (Bond) Angle(s) in CIF ...      37.40 Deg.
              C5   -C2   -H2AB    1_555   1_555   1_555 ......     #  643 Check
PLAT779_ALERT_4_G Suspect or Irrelevant (Bond) Angle(s) in CIF ...      44.80 Deg.
              C9   -C4   -C7      1_555   1_555   1_555 ......     #  656 Check
PLAT779_ALERT_4_G Suspect or Irrelevant (Bond) Angle(s) in CIF ...      41.30 Deg.
              N6   -N1   -C7      1_555   1_555   1_555 ......     #  664 Check
PLAT779_ALERT_4_G Suspect or Irrelevant (Bond) Angle(s) in CIF ...      39.50 Deg.
              C7   -C9   -N6      1_555   1_555   1_555 ......     #  670 Check
PLAT779_ALERT_4_G Suspect or Irrelevant (Bond) Angle(s) in CIF ...      36.30 Deg.
              N1   -N6   -C9      1_555   1_555   1_555 ......     #  681 Check
PLAT779_ALERT_4_G Suspect or Irrelevant (Bond) Angle(s) in CIF ...      44.30 Deg.
              C9   -C7   -C4      1_555   1_555   1_555 ......     #  685 Check
PLAT779_ALERT_4_G Suspect or Irrelevant (Bond) Angle(s) in CIF ...      38.20 Deg.
              C9   -C7   -N1      1_555   1_555   1_555 ......     #  686 Check
PLAT779_ALERT_4_G Suspect or Irrelevant (Bond) Angle(s) in CIF ...      26.80 Deg.
              C045 -C10  -H10A    1_555   1_555   1_555 ......     #  718 Check
PLAT789_ALERT_4_G Atoms with Negative _atom_site_disorder_group  #         21 Check
PLAT860_ALERT_3_G Number of Least-Squares Restraints .............       2255 Note
PLAT909_ALERT_3_G Percentage of I>2sig(I) Data at Theta(Max) Still        60% Note
PLAT933_ALERT_2_G Number of HKL-OMIT Records in Embedded .res File          3 Note
PLAT941_ALERT_3_G Average HKL Measurement Multiplicity ...........        3.6 Low
PLAT978_ALERT_2_G Number C-C Bonds with Positive Residual Density.          5 Info
```

---

0 **ALERT level A** = Most likely a serious problem - resolve or explain
0 **ALERT level B** = A potentially serious problem, consider carefully
23 **ALERT level C** = Check. Ensure it is not caused by an omission or oversight
89 **ALERT level G** = General information/check it is not something unexpected

3 ALERT type 1 CIF construction/syntax error, inconsistent or missing data
33 ALERT type 2 Indicator that the structure model may be wrong or deficient
9 ALERT type 3 Indicator that the structure quality may be low
66 ALERT type 4 Improvement, methodology, query or suggestion
1 ALERT type 5 Informative message, check

It is advisable to attempt to resolve as many as possible of the alerts in all categories. Often the minor alerts point to easily fixed oversights, errors and omissions in your CIF or refinement strategy, so attention to these fine details can be worthwhile. In order to resolve some of the more serious problems it may be necessary to carry out additional measurements or structure refinements. However, the purpose of your study may justify the reported deviations and the more serious of these should normally be commented upon in the discussion or experimental section of a paper or in the "special_details" fields of the CIF. checkCIF was carefully designed to identify outliers and unusual parameters, but every test has its limitations and alerts that are not important in a particular case may appear. Conversely, the absence of alerts does not guarantee there are no aspects of the results needing attention. It is up to the individual to critically assess their own results and, if necessary, seek expert advice.

Publication of your CIF in IUCr journals

A basic structural check has been run on your CIF. These basic checks will be run on all CIFs submitted for publication in IUCr journals (*Acta Crystallographica*, *Journal of Applied Crystallography*, *Journal of Synchrotron Radiation*); however, if you intend to submit to *Acta Crystallographica Section C* or *E* or *IUCrData*, you should make sure that full publication checks are run on the final version of your CIF prior to submission.

Publication of your CIF in other journals

Please refer to the *Notes for Authors* of the relevant journal for any special instructions relating to CIF submission.

PLATON version of 18/05/2022; check.def file version of 17/05/2022

Datablock wky - ellipsoid plot

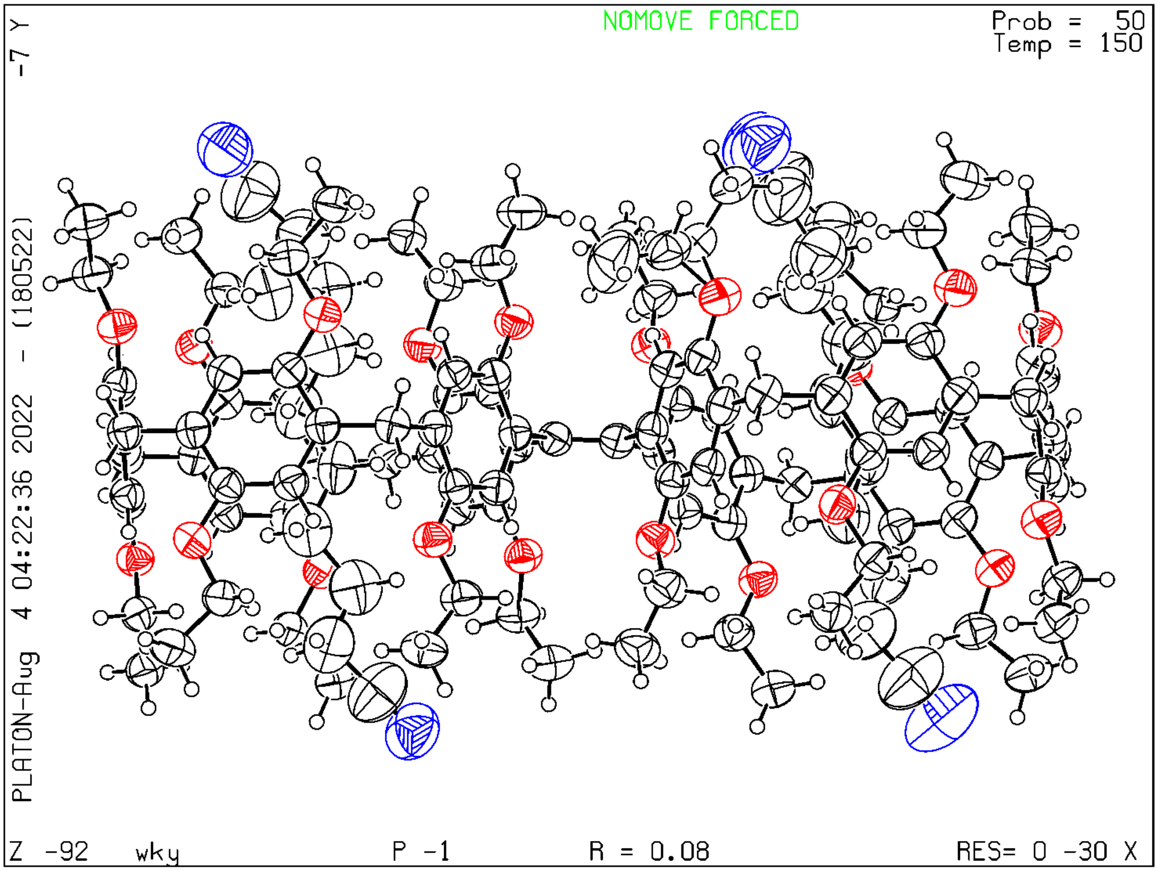

Supplement: Supplementary file 2 — Supporting Information [file ADVS-10-2206897-s002.zip › EtP5 Dimer-G2.pdf]
